# Towards potential nanoparticle contrast agents: Synthesis of new functionalized PEG bisphosphonates

**DOI:** 10.3762/bjoc.12.130

**Published:** 2016-07-04

**Authors:** Souad Kachbi-Khelfallah, Maelle Monteil, Margery Cortes-Clerget, Evelyne Migianu-Griffoni, Jean-Luc Pirat, Olivier Gager, Julia Deschamp, Marc Lecouvey

**Affiliations:** 1 Université Paris 13, Sorbonne Paris Cité, Laboratoire de Chimie, Structure, Propriétés de Biomatériaux et d’Agents Thérapeutiques (CSPBAT), CNRS UMR 7244, F-93017 Bobigny, France; 2 ICG Montpellier-UMR 5253, Equipe AM2N, ENSCM, 8, Rue de l’Ecole Normale, F-34296 Montpellier Cedex 5, France

**Keywords:** bisphosphonate, Fe_2_O_3_ nanoparticle, polyethylene glycol derivatives, surface ligand synthesis

## Abstract

The use of nanotechnologies for biomedical applications took a real development during these last years. To allow an effective targeting for biomedical imaging applications, the adsorption of plasmatic proteins on the surface of nanoparticles must be prevented to reduce the hepatic capture and increase the plasmatic time life. In biologic media, metal oxide nanoparticles are not stable and must be coated by biocompatible organic ligands. The use of phosphonate ligands to modify the nanoparticle surface drew a lot of attention in the last years for the design of highly functional hybrid materials. Here, we report a methodology to synthesize bisphosphonates having functionalized PEG side chains with different lengths. The key step is a procedure developed in our laboratory to introduce the bisphosphonate from acyl chloride and tris(trimethylsilyl)phosphite in one step.

## Introduction

Numerous researchers are interested in the development of superparamagnetic iron oxide nanoparticles (SPIONPs) because of their biocompatibility which allows there in vivo use both for diagnosis in magnetic resonance imaging and in therapy [1–2]. Most often, it is necessary to modify the surface of SPIONPs to increase the metabolic stability.

To overcome this main drawback, the NP surface could be derivatized by various functional groups. These ligands have to possess certain chemical and biological properties as the flexibility, the hydrophilicity and an absence of in vivo toxicity. In addition, the nanoparticulate systems so obtained must be stable in the various biological compartments and they must be stealthy to avoid the elimination by macrophages.

**Figure 1:**
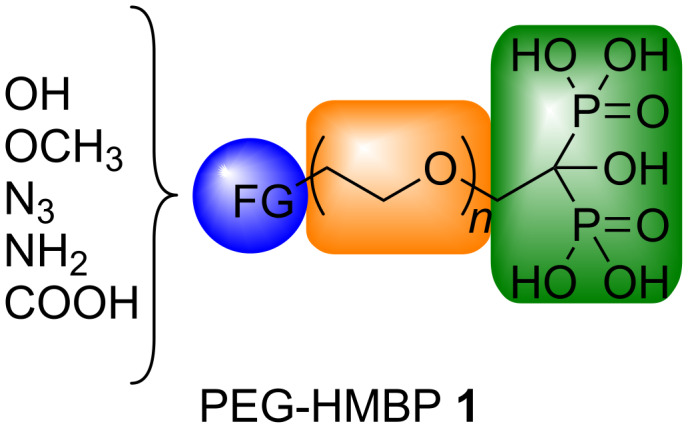
Bifunctional PEG-HMBPs **1**.

For this purpose, appropriate coatings have already been reported [3–4]. Some of which consist in the NP surface modiﬁcation using hydrophilic polymers (dextran, PEG) or bifunctional molecules substituted by amines, thiols, carboxylates, sulfonates, phosphonates or bisphosphonates [5–7]. Particularly, a strong interaction between the NPs and the phosphonic moiety was observed and more interestingly the best results were obtained with bisphosphonate products [8–9]. For the past years, our group has focused its interest in the synthesis of various functionalized hydroxymethylene bisphosphonates (HMBPs) [10] and their applications in health science, especially in antitumor therapy [11–13]. Herein, we described the synthesis of novel bifunctional PEG-HMBP compounds in order to employ them as anchoring agents for SPIONPs (Figure 1).

## Results and Discussion

**Scheme 1:**
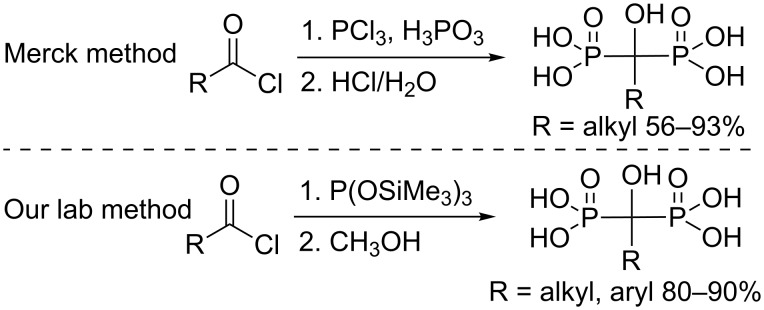
Direct methods for the 1-hydroxyalkylidenebisphosphonic acid synthesis.

For this family of compounds, the HMBP moiety has to be built starting from a modified PEG chain. The HMBP introduction could be achieved by several reported methodologies starting from an acid chloride (Scheme 1).

The first method, used in the industry, allows accessing the desired products in one step under rather harsh conditions [14]. 1-Hydroxyalkylidenebisphosphonic acids have also been obtained in good yields. However, this widely used method seems not to be compatible with breakable and delicate functionalized substrates. In contrast, our lab has developed a new synthetic strategy starting from an acid chloride and tris(trimethylsilyl) phosphite, followed by a methanolysis step [15].

This one-pot procedure allows the synthesis of various aliphatic and aromatic bisphosphonic acids under mild conditions. Moreover, reactions were very fast and pure products were obtained after evaporation of the volatile fraction. The scope of this reaction was successfully widened in aliphatic and aromatic anhydride [15–21]. The introduction of the HMBP moiety in presence of the PEG tether seems to be critical due to its high sensitivity under harsh conditions. Wherefore, our methodology, which exhibits a high tolerance to various functionalized groups, appears to be an adequate way to introduce the HMBP chain in presence of the PEG moiety.

**Scheme 2:**
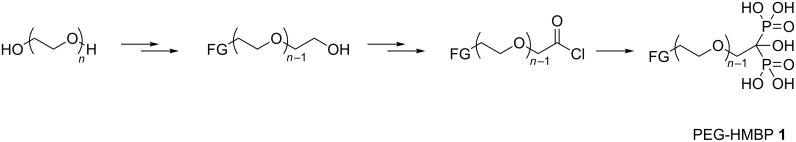
Synthetic strategy of PEG-HMBPs **1**.

To obtain the PEG-HMBP **1** compound family, the synthetic strategy consists in mono-protecting and/or mono-functionalizing commercially available PEGs followed by the lab-made HMBP methodology introduced previously (Scheme 2).

**Scheme 3:**
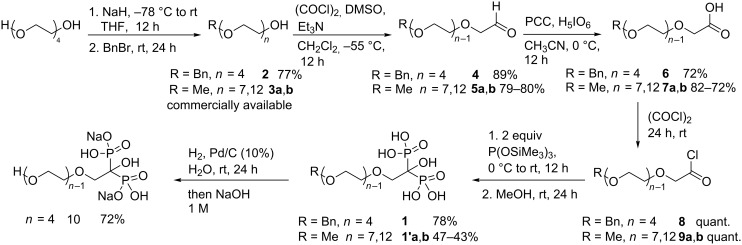
Synthesis of PEG-HMBPs **1** and **1’**.

Starting materials, the free alcohol PEG and monomethyl ether PEGs (compounds **3a**,**b**) with various chain lengths (*n* = 4, 7 and 12) were commercially available. Firstly, the free alcohol PEG was selectively monoprotected with a benzyl group (Scheme 3).

Only one alcohol function was indeed deprotonated with one equivalent of sodium hydride at −78 °C in THF after the solution was stirred for 12 hours at room temperature. The alcoholate intermediate reacted smoothly with benzyl bromide at room temperature to afford the monobenzylated PEG **2** in 77% yield (Table 1, entry 1). Afterwards, the alcohols **2** and **3a**,**b** have to be oxidized to the corresponding carboxylic acids **6** and **7a**,**b**. First of all, the direct oxidation reported in the literature in one step has been performed. Thus, tested oxidants were the Jones reagent [22], potassium permanganate [23], with catalytic *o*-iodoxybenzoic acid (IBX) in oxone [24] and catalytic 2,2,6,6-tetramethyl-1-piperidinyloxy (TEMPO) with bis(acetoxy)iodobenzene (BAIB) [25]. The first two conditions led to a PEG chain cleavage and the recovery of benzoic acid from alcohol **2**. Besides, the mixture IBX/oxone gave the expected product inseparable of IBX byproducts. Only oxidation using TEMPO and BAIB furnished the pure corresponding carboxylic acid. Nevertheless, the low obtained yields encouraged us to test a strategy in two steps via an aldehyde. Fortunately, the following two-step procedure was more effective. The alcohol derivatives **2** and **3a**,**b** were treated with dimethyl sulfoxide, oxalyl chloride and triethylamine in dichloromethane at −55 °C. Under these classical Swern conditions, the corresponding products **4** and **5a**,**b** were isolated in excellent yields from 79% to 89% (Table 1, entries 2–4). The aldehydes **4** and **5a**,**b** were next smoothly oxidized in the presence of a catalytic amount of PCC and a the co-oxidative agent H_5_IO_6_ in acetonitrile at 0 °C. The PEG **6** and **7a**,**b** were obtained in good yields (Table 1, entries 5–7).

**Table 1:** Synthesis of PEG-HMBPs **1**,**1’**and **10**.

Entry	Compound	R	*n*	Yield (%)	^31^P δ (ppm)

1	**2**	Bn	4	77	–
2	**4**	Bn	4	89	–
3	**5a**	Me	7	79	–
4	**5b**	Me	12	80	–
5	**6**	Bn	4	72	–
6	**7a**	Me	7	82	–
7	**7b**	Me	12	72	–
8	**8**	Bn	4	quant.	–
9	**9a**	Me	7	quant.	–
10	**9b**	Me	12	quant.	–
11	**1**	Bn	4	78	16.8
12	**1’a**	Me	7	47	17.2
13	**1’b**	Me	12	43	17.2
14	**10**	H	4	72	16.2

^a^Isolated yield. ^b^proton decoupling ^31^P NMR experiment.

Finally, the optimized two-step procedure enabled us to isolate the expected carboxylic acids **6** and **7a**,**b** which are key intermediates in the synthesis of PEG-HMBPs.

The carboxylic acids **6** and **7a**,**b** reacted quantitatively with oxalyl chloride to give acyl chlorides **8** and **9a**,**b** at room temperature after 24 hours (Table 1, entries 8–10). The completion of the reaction was monitored by infrared spectroscopy with the disappearance of the hydroxy absorption band and the shifting of the carbonyl vibration band to about 1800 cm^−1^. The addition of two equivalents of tris(trimethylsilyl) phosphite to the acyl chloride derivatives **8** and **9a**,**b** yielded the corresponding silylated PEG-HMBP.

The formation of silylated bisphosphonate was monitored by ^31^P NMR. After evaporation of volatile compounds under vacuum the silylated PEG-HMBP was hydrolyzed with methanol at room temperature for 24 hours. After methanol evaporation, the crude PEG-HMBP containing phosphorous acid was purified by successive washes with dry diethyl ether. The pure targeted PEG-HMBPs **1** and **1’a**,**b** were then obtained in moderate yields (Table 1, entries 11–13). The treatment of **1** with dihydrogen and palladium on charcoal in water allowed cleavage of the benzyl moiety and led to the HO-PEG-HMBP **10** in 72% yield (Table 1, entry 14). The ligand **10** permitted to obtain new gadolinium phosphate nanocrystals with luminescent properties [26].

**Scheme 4:**
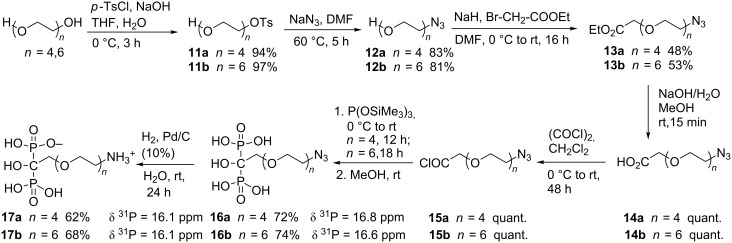
Syntheses of HMBP-PEG-N_3_ **16** and HMBP-PEG-NH_3_^+ ^**17**.

Therefore, we considered the syntheses of other compounds which possess azido or amino functional groups. The azido products will allow us to perform click chemistry to introduce various functionalities. Moreover, the amino derivatives will be easily obtained by reducing the azido group. As previously mentioned, the first step was a selective mono-activation of PEG using *para-*toluenesulfonyl chloride in the presence of sodium hydroxide in a water/THF mixture (Scheme 4).

The tosylated products **11a**,**b** were obtained after three hours at 0 °C with a 95% yields. Next, the monoactivated compounds **11a**,**b** were substituted by sodium azide in DMF at 60 °C within five hours. The azido compounds **12a**,**b** were obtained in 83% and 81% yield, respectively. The alcohols **12a**,**b** were subsequently firstly deprotonated with NaH in DMF and the generated alcoholates were stirred 16 hours with ethyl bromoacetate giving the expected esters **13a**,**b** in moderate yields.

The saponification reactions of the esters **13a**,**b** were carried out with sodium hydroxide in methanol. The completion of the reactions was controlled by TLC. After neutralization with a cationic exchange Dowex^®^ 50WX2 resin, the corresponding carboxylic acids **14a**,**b** were isolated in quantitative yields. The HMBP moiety was subsequently incorporated by the lab-made methodology previously described. The carboxylic acids **14a**,**b** were converted into the expected HMBP-PEG-N_3_ **16a**,**b** via the corresponding acyl chloride **15a**,**b**. The reactions were monitored by ^31^P NMR, compounds **16a** and **16b** were obtained after 12 and 18 hours, respectively.

Thus, HMBP-PEG-N_3_ **16a**,**b** were obtained after purification in 72% and 74% yield and characterized by a singlet in ^31^P [27] NMR at about 17 ppm. Finally, the reduction of the azido compounds **16a**,**b** in the presence of palladium on charcoal and dihydrogen led to the targeted amino-PEG-HMBPs **17a** and **17b**, respectively in moderate 62% and 68% yields.

**Scheme 5:**
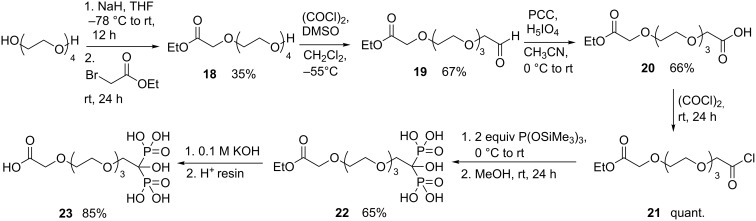
Synthesis of HMBP-PEG-COOH **23**.

In order to access available PEG-HMBPs functionalized with a primary amine or a carboxylic acid group usable in peptidic coupling with various molecules for example, the HMBP-PEG-COOH **23** was synthesized (Scheme 5). This compound was obtained in six steps starting from a free alcohol four-unit PEG. It reacted with ethyl bromoacetate after mono-deprotonation using sodium hydride in THF at −78 °C for 12 hours at room temperature giving PEG **18**. HMBP-PEG **22** was next synthesized in four steps with satisfying yields in a similar strategy previously described for compounds **1** and **1’a**,**b**. The last step was the saponification of the ethyl ester group. Different usual conditions were tested, leading to partial degradation of the HMBP. The use of a diluted aqueous solution of potassium hydroxide (0.1 M) followed by a protonation with a Dowex^®^ 50WX2 H^+^ resin allowed us to obtain the expected HMBP-PEG-COOH **23** in 85% yield.

## Conclusion

In summary, novel bifunctional PEG-HMBPs ligands for the coating of iron oxide nanoparticles have been synthesized. The procedure is efficient to introduce different functional groups such as azide, carboxylic acid, amine permitting further coupling reactions with a drug, protein or antibody. The use of PEG polymers with chains of different lengths has also given satisfying results. This modulation would allow improving the nanoparticles stealth in vivo. Further studies in this area and their applications will be reported in due course.

## Supporting Information

File 1 Experimental and analytical data of all new compounds as well as copies of their ^1^H, ^31^P and ^13^C NMR spectra.
