# Supplementary material for: Towards potential nanoparticle contrast agents: Synthesis of new functionalized PEG bisphosphonates
Source: Beilstein J Org Chem. 2016 Jul 4;12:1366–71. doi: 10.3762/bjoc.12.130 (PMC4979661; doi:10.3762/bjoc.12.130)
Supplement: File 1 — Experimental and analytical data of all new compounds as well as copies of their 1H, 31P and 13C NMR spectra. [file Beilstein_J_Org_Chem-12-1366-s001.pdf]

Supporting Information

for

Towards potential nanoparticles contrast agents: Synthesis of new functionalized PEG bisphosphonates

Souad Kachbi-Khelfallah[1], Maelle Monteil[1], Margery Cortes-Clerget[1], Evelyne Migianu-Griffoni[1], Jean-Luc Pirat[2], Olivier Gager[1], Julia Deschamp[1] and Marc Lecouvey*[1]

Address: [1]Université Paris 13, Sorbonne Paris Cité, Laboratoire de Chimie, Structure, Propriétés de Biomatériaux et d'Agents Thérapeutiques (CSPBAT), CNRS UMR 7244, F-93017 Bobigny, France and [2]ICG Montpellier-UMR 5253, Equipe AM2N, ENSCM, 8, Rue de l'Ecole Normale, F-34296 Montpellier Cedex 5, France

Email: Marc Lecouvey - marc.lecouvey@univ-paris13.fr

*Corresponding author

Experimental and analytical data of all new compounds as well as copies of their ^{1}H, ^{31}P and ^{13}C NMR spectra

General

All reactions were carried out under an argon atmosphere. All starting materials were purchased from commercial sources and used without any

further purification. More particularly, compounds **3a**, **3b**, PEG 200 alcohol and Dowex® 50WX2 H^+ resins were purchased by Sigma-Aldrich. Bisphosphonate **10** was synthesized as reported in the literature [1].

Infrared spectra were recorded on a Thermo Electron Corporation Nicolet 380 FT-IR spectrometer between 4000 and 500 cm^{-1} (16 scans, resolution = 1 to 2 cm^{-1}). The samples were analyzed as films placed between two pieces of KBr for oils.

High resolution mass spectrometry spectra were acquired using a WATERS Micromass Q-tof spectrometer in positive mode (ES+).

NMR spectra were recorded on a Bruker AVANCE III 400 MHz spectrometer. Chemical shifts (δ) and coupling constants (*J*) were expressed in ppm and Hz respectively. All compounds were analysed in D_2O or $CDCl_3$ solvents. The chemical shifts of ^1H were reported in delta (δ) units, parts per million (ppm). Chemical shifts of ^{13}C were reported in delta (δ) units. Chemical shifts of ^{31}P were reported in delta (δ) units, ppm downfield from H_3PO_4 (0.0 ppm). NMR multiplicities were abbreviated as follows: s = singlet, d = doublet, t = triplet, q = quartet, qt = quintuplet, m = multiplet, br = broad signal. All ^{31}P spectra were realized with ^1H decoupling.

The analytical data for the known compounds were found to match with the literature data.

General procedure for the preparation of bisphosphonic acid compounds as described in literature [2, 3] (1, 1'a,b, 16a,b, 22).

A 25 mL three-necked flask under argon equipped with a thermometer and a dropping funnel was charged with the adequate acid chloride **8, 9a,b**, **15a,b** or

21 (5 mmol). Then, tris(trimethylsilyl) phosphite (2.98 g, 10 mmol) was added dropwise at 0 °C. Once the addition was complete, the reaction mixture was stirred at room temperature. The evolution of the reaction was monitored by ^{31}P [4] NMR. Then, volatile fractions were evaporated under reduced pressure (0.1 Torr) before being hydrolyzed with methanol. After evaporation, crude products were obtained as an oil.

*(3,6,9,12-Tetraoxa-13-phenyl-1-hydroxytridecane-1,1-diyl)bisphosphonic acid (**1**)*. Brown oil; yield = 78%; δ_H (400 MHz, D_2O) 7.55-7.08 (5H, m, C_6H_5), 4.54 (2H, s, $C_6H_5CH_2$), 4.01-3.84 (2H, m, $OCH_2C(H_2PO_3)_2OH$), 3.83-3.55 (12H, m, OCH_2CH_2); δ_C (100.6 MHz, D_2O) 137.1, 128.3-127.5, 73.4 (t, $^1J_{C\text{-}P}$ = 117.8 Hz, 72.6-69.5, 60.2; δ_P (162 MHz, D_2O) 16.8. νmax/cm^{-1} 3400, 1246, 1232, 1103, 945; HRMS (EI): *m/z* calcd. for $C_{15}H_{27}O_{11}P_2$ $[M+H]^+$: 445.1029; found: 445.1031.

*(3,6,9,12,15,18,21-Heptaoxa-1-hydroxydocosane-1,1-diyl)bisphosphonic acid (**1'a**)*. Yellow oil; yield = 47%; δ_H (400 MHz, D_2O) 3.79 (2H, s, $OCH_2C(H_2PO_3)_2OH$), 3.53-3.65 (22H, m, OCH_2CH_2), 3.50-3.54 (2H, m, CH_2OCH_3), 3.35 (3H, s, OCH_3); δ_C (100.6 MHz, D_2O) 73.4 (t, $^1J_{C\text{-}P}$ = 101.0 Hz, P-C-P), 70.9, 70.1-68.7, 58.9; δ_P (162 MHz, D_2O) 17.2. νmax/cm^{-1} 3400, 1246, 1103, 945; HRMS (EI): *m/z* calcd. for $C_{15}H_{35}O_{14}P_2$ $[M+H]^+$: 501.1502; found: 501.1503.

*(3,6,9,12,15,18,21,24,27,30,33,36-Dodecaoxa-1-hydroxy-heptatriacontane-1,1-diyl)bisphosphonic acid (**1'b**)*. Brown oil; yield = 43%; δ_H (400 MHz, D_2O) 3.50-4.00 (50H, m, OCH_2CH_2, $OCH_2C(H_2PO_3)OH$, CH_2OCH_3), 3.31 (3H, s, OCH_3); δ_C (100.6 MHz, D_2O) 77.2 (t, $^1J_{C\text{-}P}$ = 101.0 Hz, P-C-P), 70.7-70.2, 59.1;

δ_P (162 MHz, D_2O) 17.2; νmax/cm^{-1} 3389, 1265, 1019, 951; HRMS (EI): *m/z* calcd. for $C_{25}H_{55}O_{19}P_2$ [M+H]$^+$: 721.2813; found: 721.2817.

*(14-Azido-1-hydroxy-3,6,9,12-tetraoxatetradecane-1,1-diyl)bisphosphonic acid (**16a**)*. Yellow oil; yield = 72%; δ_H (400 MHz, D_2O) 3.75-3.52 (16H, m, OCH_2CH_2), 3.46-3.37 (2H, m, CH_2N_3); δ_C (100.6 MHz, D_2O) 71.4 (t, $^1J_{C-P}$ = 137.1 Hz, P-C-P), 69.9-69.5, 63.5, 52.5; δ_P (162 MHz, D_2O) 16.8; νmax/cm^{-1} 2132, 1087, 947; HRMS (EI): *m/z* calcd. for $C_{10}H_{24}N_3O_{11}P_2$ [M+H]$^+$: 424.0886 found: 424.0884.

*(20-Azido-1-hydroxy-3,6,9,12,15,18-hexaoxaicosane-1,1-diyl)bisphosphonic acid (**16b**)*. Yellow oil; yield = 74%; δ_H (400 MHz, D_2O) 3.88-3.78 (2H, m, $OCH_2C(H_2PO_3)_2OH$), 3.74-3.52 (22H, m, OCH_2CH_2), 3.49-3.39 (2H, m, CH_2N_3); δ_C (100.6 MHz, D_2O) 71.2 (t, $^1J_{C-P}$ = 136.3 Hz, P-C-P), 69.9-69.4, 63.4, 52.5; δ_P (162 MHz, D_2O) 16.6; νmax/cm^{-1} 2132, 1087, 947; HRMS (EI): *m/z* calcd. for $C_{14}H_{32}N_3O_{13}P_2$ [M+H]$^+$: 512.1404; found: 512.1400.

*(1-Hydroxy-14-oxo-3,6,9,12,15-pentaoxaheptadecane-1,1-diyl)bisphosphonic acid (**22**)*. Brown oil; yield = 65%; δ_H (400 MHz, D_2O) 4.19-4.06 (4H, m, $OCH_2COOCH_2CH_3$), 3.82-3.21 (14H, m, OCH_2CH_2), 1.14 (3H, t, J = 8 Hz, CH_3); δ_C (100.6 MHz, D_2O) 170.5, 72.2, 70.4, 70.2, 69.5, 67.6, 52.5, 14.4; δ_P (162 MHz, D_2O) 15.7; νmax/cm^{-1} 3400, 1746, 1210, 955; HRMS (EI): *m/z* calcd. for $C_{25}H_{55}O_{19}P_2$ [M+H]$^+$: 721.2813; found: 721.2817.

General procedure for the preparation of protected compounds analogously to the description in literature [2](2, 18).

A dry and argon flushed 125 mL four-necked flask equipped with a mechanical stirrer, a thermometer, an argon inlet and an addition funnel, was charged with

sodium hydride (1.3 g, 56.1 mmol) and anhydrous THF (40 mL). Then, a solution of PEG (10.2 g, 51 mmol) in anhydrous THF (60 mL) was added at –78 °C and the reaction mixture was stirred at room temperature overnight. Then, benzyl bromide for **2** (6.1 mL, 51 mmol) or ethyl bromoacetate for **18** (6.1 mL, 51 mmol) was added dropwise and the resulting solution was stirred at room temperature. The reaction is monitored by TLC and ^{1}H NMR. After completion of the reaction, the reaction mixture was filtered and evaporated under reduced pressure. The residue was dissolved in dichloromethane (7 mL), washed three times with water (20 mL) and the combined organic layer was dried with $MgSO_4$ and concentrated in vacuo.

1-Phenyl-2,5,8,11-tetraoxatridecan-13-ol (***2***). (TLC eluent: 20/80 hexane/ ethyl acetate). Yellow oil; yield = 77%, δ_H (400 MHz, $CDCl_3$) 7.49-7.11 (5H, m, C_6H_5), 4.55 (2H, s, $C_6H_5CH_2$), 3.78-3.45 (16H, m, OCH_2CH_2), 3.11 (1H, s, OH); δ_C (100.6 MHz, $CDCl_3$) 138.2, 128.3-127.5, 73.1, 72.4-69.3, 61.5. νmax/cm^{-1} 3424, 3087-3005, 2867, 1495, 1454. These data are in accordance with those reported in the literature [5].

*Ethyl 14-hydroxy-3,6,9,12-tetraoxatetradecan-1-oate (**18**)*. (TLC eluent: 95/5 dichloromethane/ ethanol). Yellow oil; yield = 35%; δ_H (400 MHz, $CDCl_3$) 4.37-4.20 (4H, m, OCH_2CH_3, $HOCH_2$), 4.17 (2H, s, $OCH_2COOCH_2CH_3$), 3.80-3.61 (16H, m, OCH_2CH_2), 1.30 (3H, t, J = 8 Hz, CH_3); δ_C (100.6 MHz, $CDCl_3$) 170.5, 70.8-70.2, 68.7, 61.7, 14.2. These data are in accordance with those reported in the literature [6].

General procedure for the preparation of aldehyde compounds analogously to the description in literature [2](4, 5a-b, 19).

In a dry and argon flushed 100 mL four-necked flask, equipped with a mechanical stirrer, a thermometer, an argon inlet and a septum, a solution of DMSO (1.7 g, 21.8 mmol) in 4.4 mL of dichloromethane was added dropwise at –55 °C to a solution of oxalyl chloride (2.16 g, 17.2 mmol) in 22.6 mL of dichloromethane. After two minutes, a solution of adequate alcohol **2**, **3a**,**b** or **18** (2.84 g, 10 mmol) in 9 mL of dichloromethane was added dropwise. After stirring for 15 min, the mixture was allowed to warm to –30 °C. Then, freshly distilled triethylamine was added dropwise (5.1 g, 50.4 mmol). After 12 hours at room temperature, the mixture was quenched with 20 mL of water. The aqueous layer was extracted twice with 20 mL of dichloromethane and the combined organic layers were washed successively with aqueous HCl (20%) and $NaHCO_3$ (5%), dried with $MgSO_4$ and concentrated in vacuo.

*1-Phenyl-2,5,8,11-tetraoxatridecan-13-al (**4**)*. Yellow oil; yield = 89%; δ_H (400 MHz, $CDCl_3$) 9.58 (1H, s, CHO), 7.51-6.98 (5H, m, C_6H_5), 4.48 (2H, s, $C_6H_5CH_2$), 4.04 (2H, s, OCH_2CHO), 3.70-3.22 (12H, m, OCH_2CH_2); δ_C (100.6 MHz, $CDCl_3$) 200.4, 137.9, 127.9-127.1, 76.3, 72.2, 70.6-69.0; νmax/cm^{-1} 3075, 2832, 1707, 1598; HRMS (EI): *m/z* calcd. for $C_{15}H_{22}O_5$ [M+H]+: 283.1757; found: 283.1754.

*2,5,8,11,14,17,20-Heptaoxadocosan-22-al (**5a**)*. Brown oil; yield = 79%; δ_H (400 MHz, $CDCl_3$) 9.67 (1H, s, CHO), 4.10 (2H, s, OCH_2CHO), 3.59-3.47 (24H, m, OCH_2CH_2), 3.31 (3H, s, OCH_3); δC (100.6 MHz, $CDCl_3$) 200.9, 71.9, 71.2-70.5, 59.1; δ_P (162 MHz, $CDCl_3$) 16.1; νmax/cm^{-1} 2832, 1707, 1120; HRMS (EI): *m/z* calcd. for $C_{15}H_{31}O_8$ [M+H]+: 339.2019; found: 339.2015.

2,5,8,11,14,17,20,23,26,29,32,35-Dodecaoxaheptatriacontan-37-al (***5b***). Brown oil; yield = 80%; δ_H (400 MHz, $CDCl_3$) 9.74 (1H, s, CHO), 4.17 (2H, s,

OCH_2CHO), 3.39 (3H, s, OCH_3), 3.66-3.77 (44H, m, OCH_2CH_2); δ_C (100.6 MHz, $CDCl_3$) 200.7, 71.7, 72.0-70.2, 58.8; HRMS (EI): *m/z* calcd. for $C_{25}H_{51}O_{13}$ [M+H]+: 559.3330; found: 559.3335.

*Ethyl 14-oxo-3,6,9,12-tetraoxatetradecan-1-oate (**19**)*. Yellow oil; yield = 67%; δ_H (400 MHz, $CDCl_3$) 9.74 (1H, s, CHO), 4.05-4.19 (4H, m, $OCH_2COOCH_2CH_3$), 3.83-3.57 (14H, m, OCH_2CH_2), 1.29 (3H, t, J = 8 Hz, CH_3); δ_C (100.6 MHz, $CDCl_3$) 170.5, 71-70.4, 68.7, 60.9, 14.4; HRMS (EI): *m/z* calcd. for $C_{12}H_{23}O_7$ [M+H]+: 280.1522; found: 280.1518.

General procedure for the preparation of acid compounds as described in literature [2] (6, 7a,b, 20).

A three necked 500 mL flask equipped with a dropping funnel was charged with 130 mL of dry acetonitrile and periodic acid H_5IO_6 (4.035 g, 17.7 mmol) and the resulting mixture was stirred at room temperature for 15 minutes. Then, a solution of the aldehyde derivative (16.1 mmol) in 16 mL of dried acetonitrile was added dropwise at 0 °C, followed by dropwise addition of a solution of pyridinium chlorochromate (0.07 g, 0.325 mmol) in 16 mL of acetonitrile. The reaction was monitored by TLC (eluent: CH_2Cl_2/EtOH = 95:5). When the reaction was complete, 40 mL of ethyl acetate were added to the mixture and the resulting solution was washed with saturated NaCl, saturated $NaHSO_3$, saturated NaCl, dried with $MgSO_4$ and concentrated in vacuo.

*13-Phenyl-3,6,9,12-tetraoxatridecan-1-oic acid (**6**)*. Brown oil; yield = 72%; δ_H (400 MHz, $CDCl_3$) 7.46-7.1 (5H, m, C_6H_5), 6.97 (1H, s, COOH), 4.57 (2H, s, $C_6H_5CH_2$), 4.21-4.00 (2H, m, OCH_2COOH), 3.79-3.56 (12H, m, OCH_2CH_2); δ_C (100.6 MHz, $CDCl_3$) 172.5, 138.1, 128.3-127.5, 73.1, 70.1-69.2, 68.5;

νmax/cm^{-1} 3062, 3029, 1757, 1352, 1110. These data are in accordance with those reported in the literature [7].

*2,5,8,11,14,17,20-Heptaoxadocosan-22-oic acid (**7a**)*. Brown oil; yield = 82%; δ_H (400 MHz, $CDCl_3$) 4.10 (2H, s, OCH_2COOH), 3.78-3.39 (24H, m, OCH_2CH_2), 3.30 (3H, s, OCH_3); δ_C (100.6 MHz, D_2O) 171.9, 70.1-71.8, 69.0, 58.9; νmax/cm^{-1} 3062, 1757, 1110. These data are in accordance with those reported in the literature [8].

*2,5,8,11,14,17,20,23,26,29,32,35-Dodecaoxaheptatriacontan-37-oic acid (**7b**)*. Brown oil; yield = 72%; δ_H (400 MHz, $CDCl_3$) 8.02 (1H, s, COOH), 4.06 (2H, s, OCH_2COOH), 3.69-3.47 (44H, m, OCH_2CH_2), 3.31 (3H, s, OCH_3); δ_C (100.6 MHz, D_2O) 171.6, 71.9, 70.5-69.0, 59.0; νmax/cm^{-1} 3058, 1746, 1122; HRMS (EI): *m/z* calcd. for $C_{25}H_{51}O_{14}$: 575.3279; found: 575.3281.

*14-Oxo-3,6,9,12,15-pentaoxaheptadecan-1-oic acid (**20**)*. Yellow oil; yield = 66%; δ_H (400 MHz, $CDCl_3$) 8.05 (1H, s, OH), 4.45 (2H, s, OCH_2COOH), 4.34-4.11 (2H, m, $OCH_2COOCH_2CH_3$), 4.10-4.07 (2H, m, $OCH_2COOCH_2CH_3$), 3.78-3.60 (14H, m, OCH_2CH_2), 1.22 (3H, t, J = 8.0 Hz, CH_3); δ_C (100.6 MHz, $CDCl_3$) 171.9, 170.6, 71.1-69.7, 69.3-68.7, 60.9, 14.4; νmax/cm^{-1} 3102, 1732-1710, 1105; HRMS (EI): *m/z* calcd. for $C_{12}H_{23}O_7$ [M+H]+: 279.1444; found: 279.1440.

General procedure for the preparation of acid chloride compounds as analogously to the description in literature [2] (8, 9a,b, 21).

A 50 mL flame-dried three-necked flask equipped with an argon inlet and a dropping funnel was charged with the carboxylic acid derivative (1 mmol) and 25 mL of freshly distilled dichloromethane. The flask was placed in ice bath (0 °C) and a solution of oxalyl chloride (0.63 g, 5 mmol) in 10 mL of

dichloromethane was added dropwise. The reaction mixture was then stirred at room temperature for 24 hours. Then, volatile fractions were co-evaporated under reduced pressure (0.01 Torr) twice with dried dichloromethane and anhydrous diethyl ether.

*13-Phenyl-3,6,9,12-tetraoxatridecan-1-oyl chloride (**8**)*. Brown oil; quantitative yield; νmax/cm^{-1} 3029, 1801, 1455, 1352, 1029, 748.

*2,5,8,11,14,17,20-Heptaoxadocosan-22-oyl chloride (**9a**)*. Brown oil; quantitative yield; νmax/cm^{-1} 1809, 1033, 1356, 751.

*2,5,8,11,14,17,20,23,26,29,32,35-Dodecaoxaheptatriacontan-37-oyl chloride (**9b**)*. Brown oil; quantitative yield; νmax/cm^{-1} 1803, 1030, 1351, 745.

*Ethyl 14-chloro-14-oxo-3,6,9,12-tetraoxatetradecan-1-oate (**21**)*. Brown oil; quantitative yield; νmax/cm^{-1} 1801, 1455, 1352, 1029, 748.

General procedure for the preparation of bisphosphonic acid compounds (17a,b).

In a 50 mL round bottom flask, the compound **16a** or **16b** (2.25 mmol) was dissolved in water (25 mL), and palladium on activated charcoal 10% (0.3 g) was added. After purging the reaction atmosphere three times with dihydrogen, the reaction mixture was stirred under H_2 atmosphere (1 atm). After 24 hours, the solution was filtered through a Celite® pad, the pH was adjusted to 6 with a solution of NaOH (1 M) and the product was freeze dried.

*(14-Ammonio-1-hydroxy-3,6,9,12-tetraoxatetradecane-1,1-diyl)bis(hydrogen phosphonate) monosodium salt (**17a**)*. Yellow solid; yield = 62%; δ_H (400 MHz, D_2O) 4.02-3.78 (2H, m, $OCH_2CH_2NH_3^+$), 3.64-3.52 (16H, m, OCH_2CH_2); δ_C (100.6 MHz, D_2O) 75.2 (t, $^1J_{C\text{-}P}$ = 116.5 Hz, P-C-P), 73.6, 69.9-69.5, 65.7, 41.4;

δ_P (162 MHz, D_2O) 16.1; νmax/cm^{-1} 3245, 1110, 1175, 966; HRMS (EI): *m/z* calcd. for $C_{10}H_{25}NO_{11}P_2Na_2$ [M+H]+: 443.0698; found: 443.0692.

*(20-Ammonio-1-hydroxy-3,6,9,12,15,18-hexaoxaicosane -1,1-diyl)bis(hydrogen phosphonate) monosodium salt (**17b**)*. Yellow solid; yield = 68%; δ_H (400 MHz, D_2O) 3.82-3.66 (2H, m, $OCH_2CH_2NH_3^+$), 3.66-3.41 (24H, m, OCH_2CH_2); δ_C (100.6 MHz, D_2O) 74.9 (t, $^1J_{C\text{-}P}$ = 116.2 Hz, P-C-P), 72.2, 71.9-69.2, 60.2, 39.8; δ_P (162 MHz, D_2O) 16.1; νmax/cm^{-1} 3237, 1102, 1169, 971; HRMS (EI): *m/z* calcd. for $C_{14}H_{33}NO_{13}P_2Na_2$ [M+H]+: 531.1223, found: 531.1219.

General procedure for the preparation of tosylate compounds (11a,b) analogously to the description in literature [9].

A solution of sodium hydroxide (5.48 g, 137 mmol) in water (30 mL) was added to a solution of PEG compound (904 mmol) in THF (30 mL). The resulting mixture was cooled to 0 °C and a solution of *p*-toluenesulfonyl chloride (16.6 g, 87.4 mmol) in THF (100 mL) was slowly added under stirring for 2 hours. After stirring at 0 °C for 3 hours, the reaction mixture was poured onto an ice/water mixture (500 mL). The organic layer was separated, and the aqueous layer was extracted with dichloromethane (3 × 200 mL). The combined organic layers were washed twice with water (100 mL), dried with $MgSO_4$ and concentrated in vacuo.

2-*(2-(2-(2-Hydroxyethoxy)ethoxy)ethoxy)ethyl-4-methylbenzenesulfonate (**11a**)*. Clear oil; yield = 94%; δ_H (400 MHz, $CDCl_3$) 7.77 (2H, d, J = 8.2 Hz, C_6H_4), 7.32 (2H, d, J = 8 Hz, C_6H_4), 4.21-4.01 (2H, m, $MeC_6H_4SO_3CH_2$), 3.79-3.47 (14H, m, OCH_2CH_2), 2.75 (1H, s, OH), 2.42 (3H, s, CH_3); δ_C (100.6 MHz, $CDCl_3$) 144.7, 132.6, 129.7, 127.7, 72.3-70.0, 69.1, 68.3, 61.3, 21.4. νmax/cm^{-1}

3468, 1295, 1103, 944, 733. These data are in accordance with those reported in the literature [10].

*17-Hydroxy-3,6,9,12,15-pentaoxaheptadecyl-4-methyl-benzenesulfonate (**11b**)*. Colorless oil; yield = 97%; δ_H (400 MHz, $CDCl_3$) 7.54 (2H, d, J = 8.3 Hz, C_6H_4), 7.13 (2H, d, J = 8.2 Hz, C_6H_4), 3.98-3.85 (2H, m, $CH_3C_6H_4SO_3CH_2$), 3.57-3.26 (22H, m, OCH_2CH_2), 2.20 (3H, s, CH_3); δ_C (100.6 MHz, $CDCl_3$) 144.3, 132.3, 129.3, 127.3, 72.0-68.9, 67.9, 60.8, 21.0. $\nu max/cm^{-1}$ 3459, 1308, 1076, 976, 751. These data are in accordance with those reported in the literature [11].

General procedure for the preparation of azide compounds (12a,b).

Sodium azide (9.34 g, 143.7 mmol) was added to a solution of the mono-tosylated PEG derivative **11a-b** (28.8 mmol) in anhydrous DMF (100 mL). The reaction mixture was then heated at 60 °C for 5 hours. The solvent was removed under reduced pressure. Water (50 mL) and dichloromethane (50 mL) were added and the aqueous layer was extracted with dichloromethane (3 × 100 mL). The combined organic layers were washed successively with water and brine, dried with $MgSO_4$ and concentrated in vacuo.

*2-{2-[2-(2-Azidoethoxy)ethoxy]ethoxy}ethanol (**12a**)*. Clear oil; yield = 83%; δ_H (400 MHz, $CDCl_3$) 3.74-3.45 (14H, m, OCH_2CH_2), 3.31-3.36 (2H, m, CH_2N_3), 2.91 (1H, s, OH); δ_C (100.6 MHz, $CDCl_3$) 72.5-69.9, 61.6, 53.5; $\nu max/cm^{-1}$ 3465, 2107, 1124. These data are in accordance with those reported in the literature [4].

*17-Azido-3,6,9,12,15-pentaoxaheptadecan-1-ol (**12b**)*. Yellow oil; yield = 81%; δ_H (400 MHz, $CDCl_3$) 3.70-3.43 (22H, m, OCH_2CH_2), 3.36-3.24 (2H, m, CH_2N_3),

3.12 (1H, s, OH); δ_C (100.6 MHz, $CDCl_3$) 72.6-69.9, 61.5, 50.6; $\nu max/cm^{-1}$ 3445, 2035, 1103. These data are in accordance with those reported in the literature [12].

General procedure for the preparation of ester compounds (13a,b) analogously to the description in literature [9].

A solution of azide **12a** or **12b** (23.8 mmol) in anhydrous DMF (7.5 mL) was added to a cooled solution of sodium hydride (0.63 g, 26.4 mmol,) in anhydrous DMF (15 mL). The resulting reaction mixture was stirred for 30 minutes and ethyl bromoacetate (2.94 mL, 26.2 mmol) was added dropwise at 0 °C. After completion of the addition, the reaction mixture was stirred for 16 hours at room temperature. After quenching with ice/water, the solvent was removed under reduced pressure. Water (50 mL) and ethyl acetate (50 mL) were added and the aqueous layer was extracted with ethyl acetate (3 × 50 mL). The combined organic layers were washed successively with water and brine, dried with $MgSO_4$ and concentrated in vacuo.

*Ethyl 14-azido-3,6,9,12-tetraoxatetradecan-1-oate (**13a**).* Brown oil; yield = 48%; δ_H (400 MHz, $CDCl_3$) 4.27-3.97 (4H, m, $OCH_2COOCH_2CH_3$), 3.75-3.48 (14H, m, OCH_2CH_2), 3.36-3.25 (2H, m, CH_2N_3), 1.31-1.07 (3H, m, CH_3); δ_C (100.6 MHz, $CDCl_3$) 170.3, 70.7-68.5, 68.3, 60.5, 50.4, 20.1, 14.1; $\nu max/cm^{-1}$ 2072, 1737, 1109. These data are in accordance with those reported in the literature [6].

*Ethyl 20-Azido-3,6,9,12,15,18-hexaoxaicosan-1-oate (**13b**).* Brown oil; yield = 53%; δ_H (400 MHz, $CDCl_3$) 4.30-4.04 (4H, m, $OCH_2COOCH_2CH_3$), 3.75-3.52 (22H, m, OCH_2CH_2), 3.44-3.31 (2H, m, CH_2N_3), 1.33-1.10 (3H, m, CH_3); δ_C

(100.6 MHz, $CDCl_3$) 170.2, 70.6-68.5, 66.6, 61.4, 50.4, 20.8; νmax/cm^{-1} 2072, 1737, 1109; HRMS (EI): *m/z* calcd. for $C_{16}H_{30}O_8N_3$[M+H]+ : 392.2033; found: 392.2031.

General procedure for the preparation of acid compounds (14a,b).

Aqueous 2 M NaOH solution (23 mL) was added to a solution of **13a** or **13b** (11.5 mmol) in methanol (23 mL). After 10 minutes, the methanol was removed under vacuum to obtain the crude product. The reaction mixture was acidified to pH = 3.5 and extracted with 3 × 30 mL of chloroform. The combined organic layers were concentrated in vacuo yielding the pure product.

*14-Azido-3,6,9,12-tetraoxatetradecan-1-oic acid (**14a**)*. Yellow oil; quantitative yield; δ_H (400 MHz, $CDCl_3$) 4.16-4.09 (2H, m, OCH_2COOH), 3.77-3.54 (14H, m, OCH_2CH_2), 3.30 (2H, t, J = 7.0 Hz, CH_2N_3); δ_C (100.6 MHz, $CDCl_3$) 172.6, 70.8-69.8, 68.4, 50.4; νmax/cm^{-1} 2009, 1740, 1115. These data are in accordance with those reported in the literature [13].

*20-Azido-3,6,9,12,15,18-hexaoxaicosan-1-oic acid (**14b**)*. Yellow oil; quantitative yield; δ_H (400 MHz, $CDCl_3$) 9.28 (1H, s, OH), 3.86 (2H, s, OCH_2COOH), 3.73-3.53 (22H, m, OCH_2CH_2), 3.47-3.32 (2H, m, CH_2N_3); δ_C (100.6 MHz, $CDCl_3$) 172.3, 70.8-69.9, 68.7, 50.6; νmax/cm^{-1} 3481, 2110, 1753, 1097. These data are in accordance with those reported in the literature [14].

General procedure for the preparation of acid chloride compounds (15a,b).

A 50 mL flame-dried three-necked flask equipped with an argon inlet and a dropping funnel was charged with the carboxylic acid derivative **14a,b** (10

mmol) and 40 mL of freshly distilled dichloromethane. The flask was placed in an ice bath (0 °C) and a solution of oxalyl chloride (6.3 g, 50 mmol) in 10 mL of dichloromethane was added dropwise. The reaction mixture was then stirred at room temperature for 48 hours. The reaction evolution was monitored by infrared spectroscopy. Then, volatile fractions were co-evaporated under reduced pressure (0.01 Torr) twice with anhydrous dichloromethane and anhydrous diethyl ether.

*14-Azido-3,6,9,12-tetraoxatetradecan-1-oyl chloride (**15a**)*. Yellow oil; quantitative yield; νmax/cm^{-1} 2109, 1802, 1130.

*20-Azido-3,6,9,12,15,18-hexaoxaicosan-1-oyl chloride (**15b**)*. Yellow oil; quantitative yield; νmax/cm^{-1} 2114, 1803, 1127.

General procedure for the preparation of 14-hydroxy-14,14-diphosphono-3,6,9,12-tetraoxatetradecan-1-oic acid (23).

The corresponding ester phosphonic compound **22** was saponified with NaOH 0.1 M solution. The solution was acidified with Dowex® 50WX2 H+ resins to pH = 1 and aqueous layer was lyophilized. Yellow solid; yield = 85%; δ_H (400 MHz, D_2O) 4.2 (2H, s, OCH_2COONa), 3.98-3.48 (14H, m, OCH_2CH_2); δ_C (100.6 MHz, D_2O) 170.5, 72.2 (t, $^1J_{C-P}$ = 115.2 Hz), 70.4, 70.2, 69.5, 67.6, 52.5, 14.4; δ_P (162 MHz, D_2O) 15.9; HRMS (EI): *m/z* calcd. for $C_{10}H_{23}O_{13}P_2$ [M+H]+: 413.0614; found: 413.0610.

References

1. Abécassis, B. ; Lerouge, F. ; Bouquet, F. ; Kachbi, S. ; Monteil, M. ; Davidson, P. *J.Phys. Chem. B*, **2012**, 116, 7590-7595.
2. Kachbi Khelfallah, S. ; Monteil, M. ; Deschamp, J. ; Gager, O. ; Migianu-Griffoni, E. ; Lecouvey, M. *Org. Biomol. Chem.*, **2015**, 13, 11382-11392.
3. Guénin, E. ; Monteil, M. ; Bouchemal, N. ; Prangé, T. ; Lecouvey, M. *Eur. J. Org. Chem.*, **2007**, 2007, 3380-3391.
4. Cubberley, M. S. ; Iverson, B. L. *J. Am. Chem. Soc.*, **2001**, 123, 7560-7563.
5. Maranski, K. ; Andreev, Y. G. ; Bruce, P. G. *Angew. Chem. Int. Ed.*, **2014**, 53, 6411-6413.
6. Bhosale, S. ; Bhosale, S. ; Wang, T. ; Kopaczynska, M. ; Fuhrhop, J.-H. *J. Am. Chem. Soc.*, **2006**, 128, 2156-2157.
7. Zhang, X. ; Du, X. ; Huang, X. ; Lv, Z. *J. Am. Chem. Soc.*, **2013**, 135, 9248-9251.
8. Kuroboshi, M. ; Yoshida, T. ; Oshitani, J. ; Goto, K. ; Tanaka, H. *Tetrahedron*, **2009**, 65, 7177-7185.
9. Shirude, P. S. ; Kumar, V. A. ; Ganesh, K. N. *Eur. J. Org. Chem.*, **2005**, 2005, 5207-5215.
10. Percec, V. ; Leowanawat, P. ; Sun, H.-J. ; Kulikov, O. ; Nusbaum, C. D. ; Tran, T. M. ; Bertin, A. ; Wilson, D. A. ; Peterca, M. ; Zhang, S. ; Kamat, N. P. ; Vargo, K. ; Moock, D. ; Johnston, E. D. ; Hammer, D. A. ; Pochan, D. J. ; Chen, Y. ; Chabre, Y. M. ; Shiao, T. C. ; Bergeron-Brlek, M. ; André, S. ; Roy, R. ; Gabius, H.-J. ; Heiney, P. A. *J. Am. Chem. Soc.*, **2013**, 135, 9055-9077.
11. Dif, A. ; Boulmedais, F. ; Pinot, M. ; Roullier, V. ; Baudy-Floc'h, M. ; Coquelle, F. M. ; Clarke, S. ; Neveu, P. ; Vignaux, F. ; Borgne, R. L. ; Dahan, M. ; Gueroui, Z. ; Marchi-Artzner, V. *J. Am. Chem. Soc.*, **2009**, 131, 14738-14746.
12. Menger, F. M. ; Zhang, H. *J. Am. Chem. Soc.*, **2006**, 128, 1414-1415.
13. Bogdan, N. D. ; Matache, M. ; Meier, V. M. ; Dobrotă, C. ; Dumitru, I. ; Roiban, G. D. ; Funeriu, D. P. *Chem. Eur. J.*, **2010**, 16, 2170-2180.
14. Gianella, A. ; Mieszawska, A. J. ; Hoeben, F. J. M. ; Janssen, H. M. ; Jarzyna, P. A. ; Cormode, D. P. ; Costa, K. D. ; Rao, S. ; Farokhzad, O. C. ; Langer, R. ; Fayad, Z. A. ; Mulder, W. J. M. *Chem. Comm.*, **2013**, 49, 9392-9394.

Ccopies of ^{1}H, ^{31}P and ^{13}C NMR spectra

(3,6,9,12-Tetraoxa-13-phenyl-1-hydroxytridecane-1,1-diyl)bisphosphonic acid (**1**).

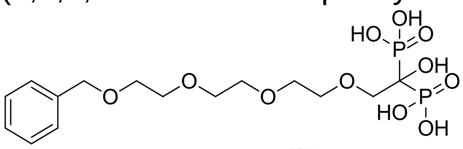

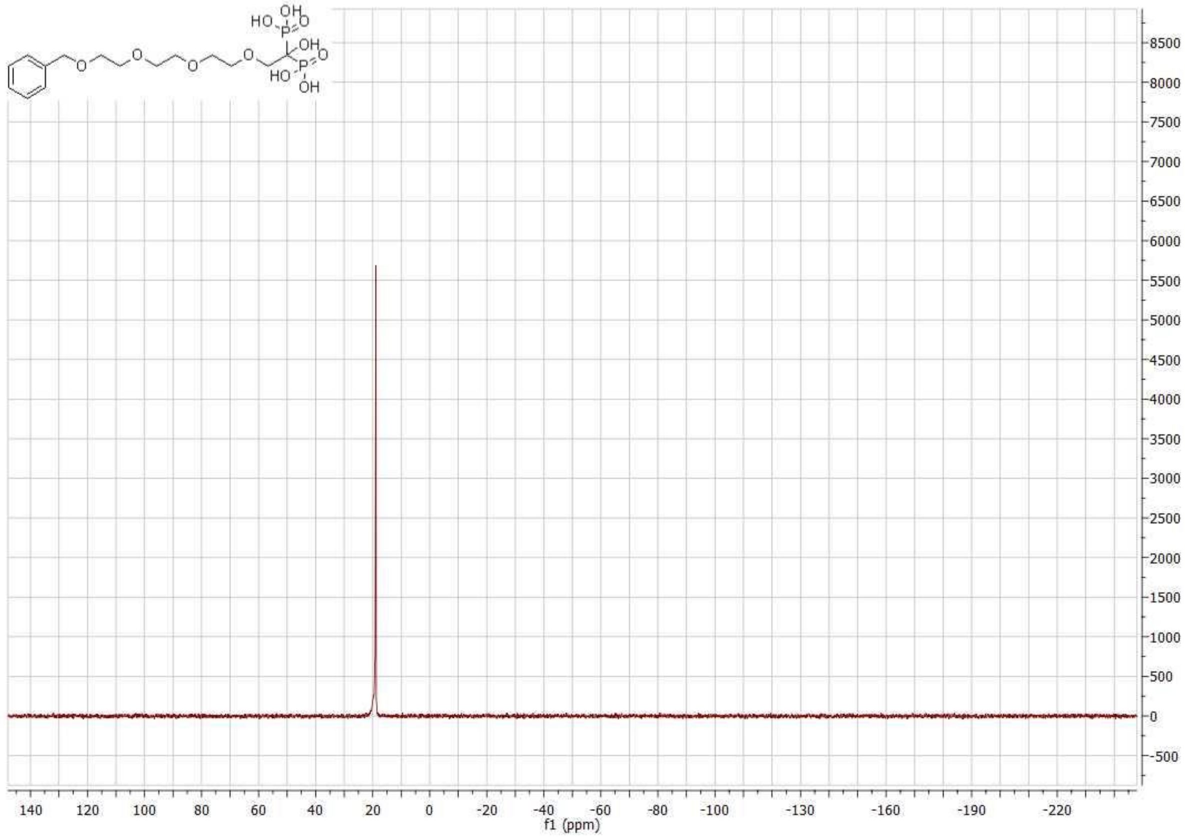

^{31} P NMR spectrum of compound **1** in D_2O

1

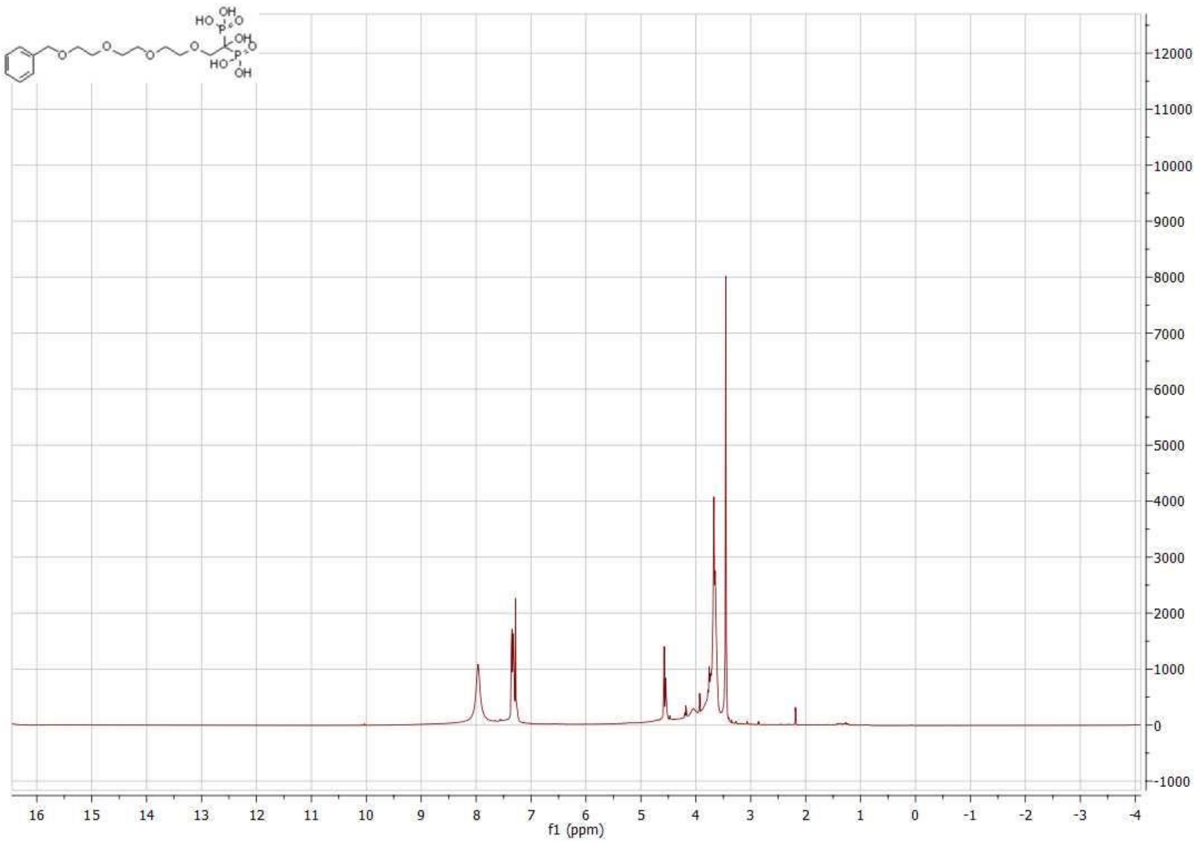

^1H NMR spectrum of compound **1** in D_2O

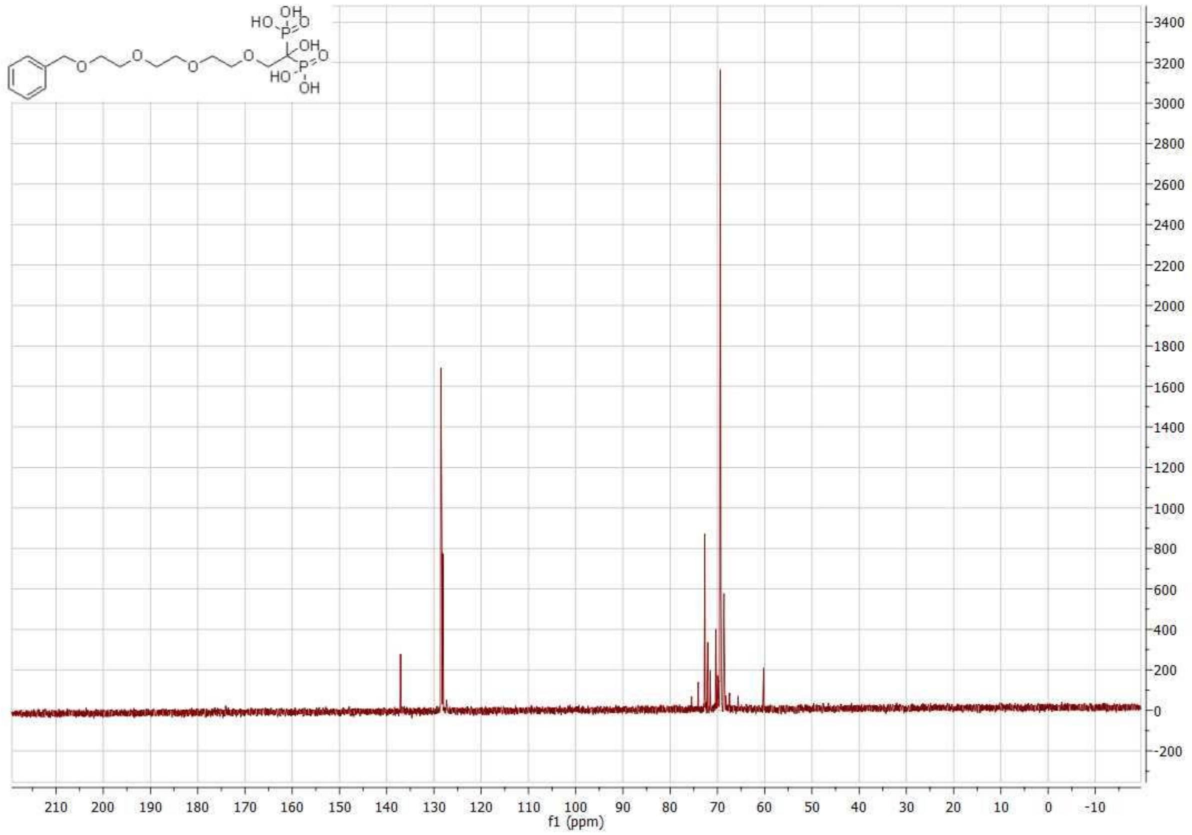

^{13}C NMR spectrum of compound **1** in D_2O

3,6,9,12,15,18,21-Heptaoxa-1-hydroxydocosane-1,1-diyl)bisphosphonic acid (**1’a**).

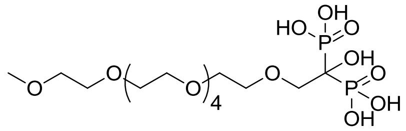

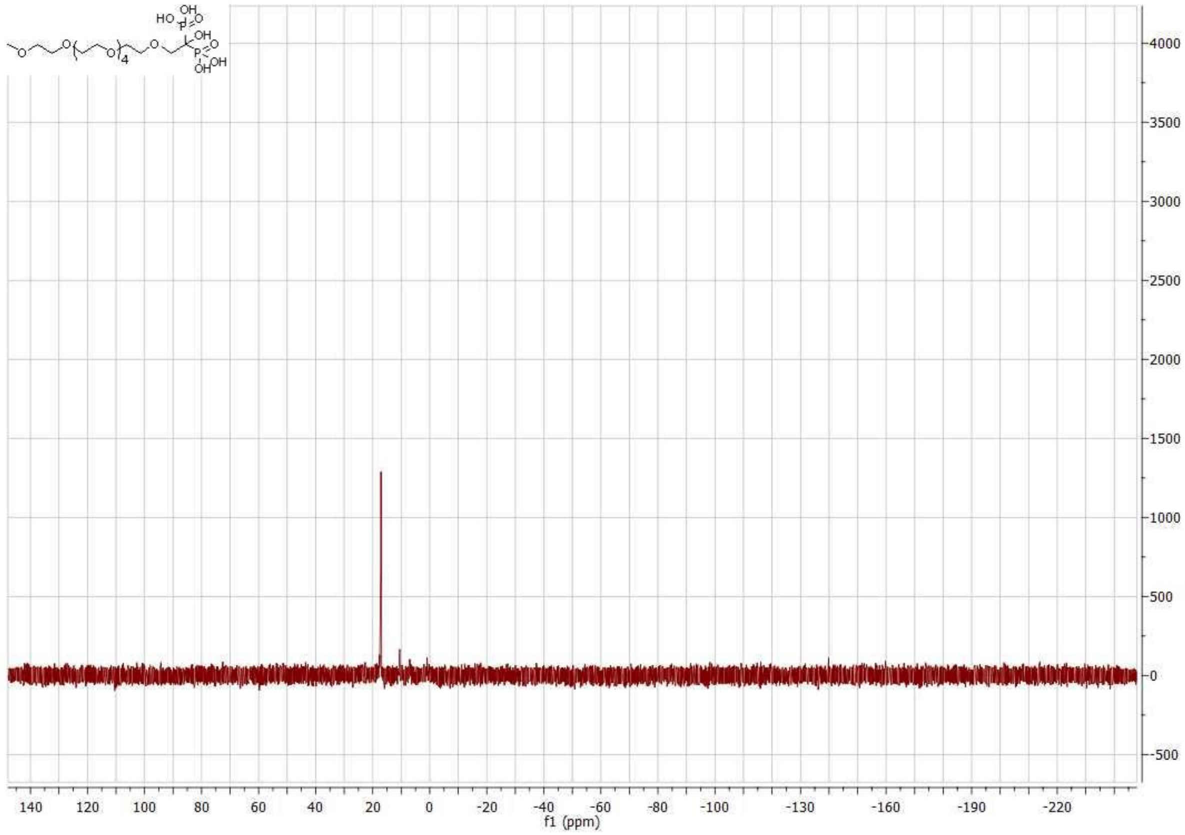

^{31}P NMR spectrum of compound **1’a** in D_2O

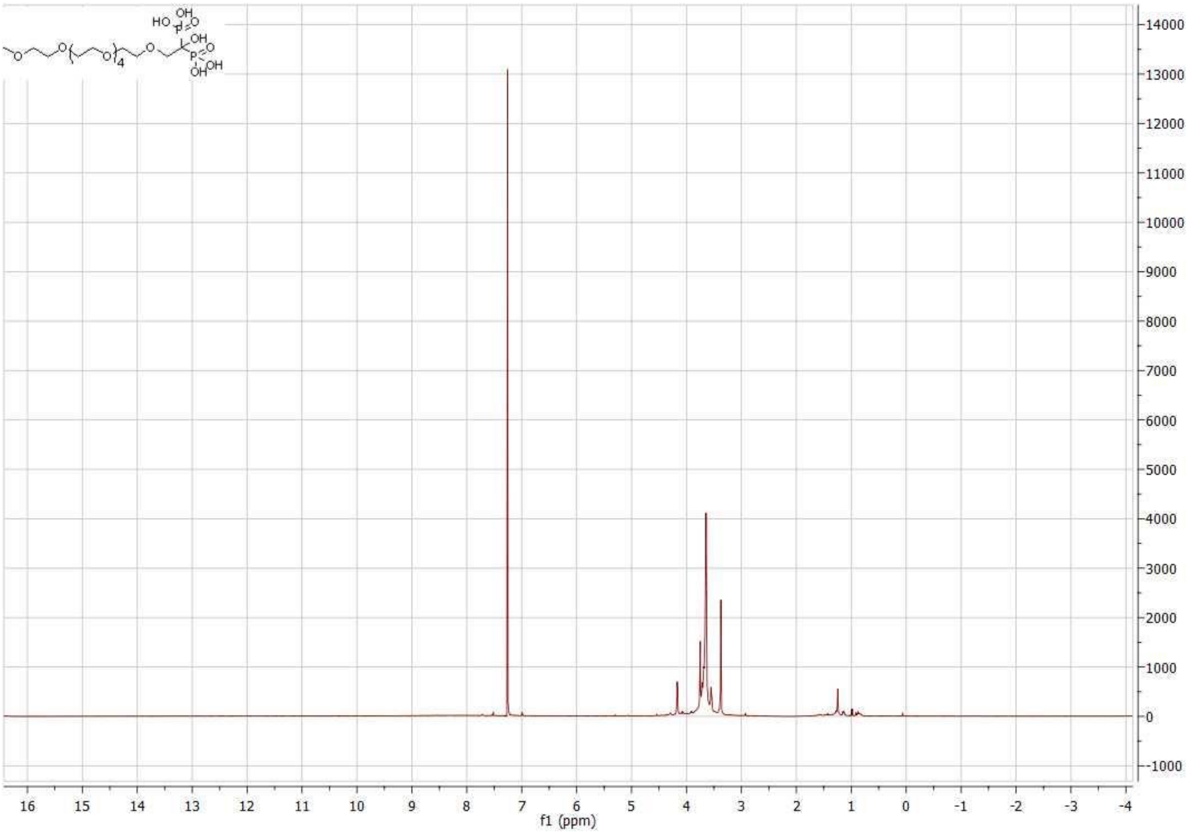

^{1}H NMR spectrum of compound **1’a** in D_2O

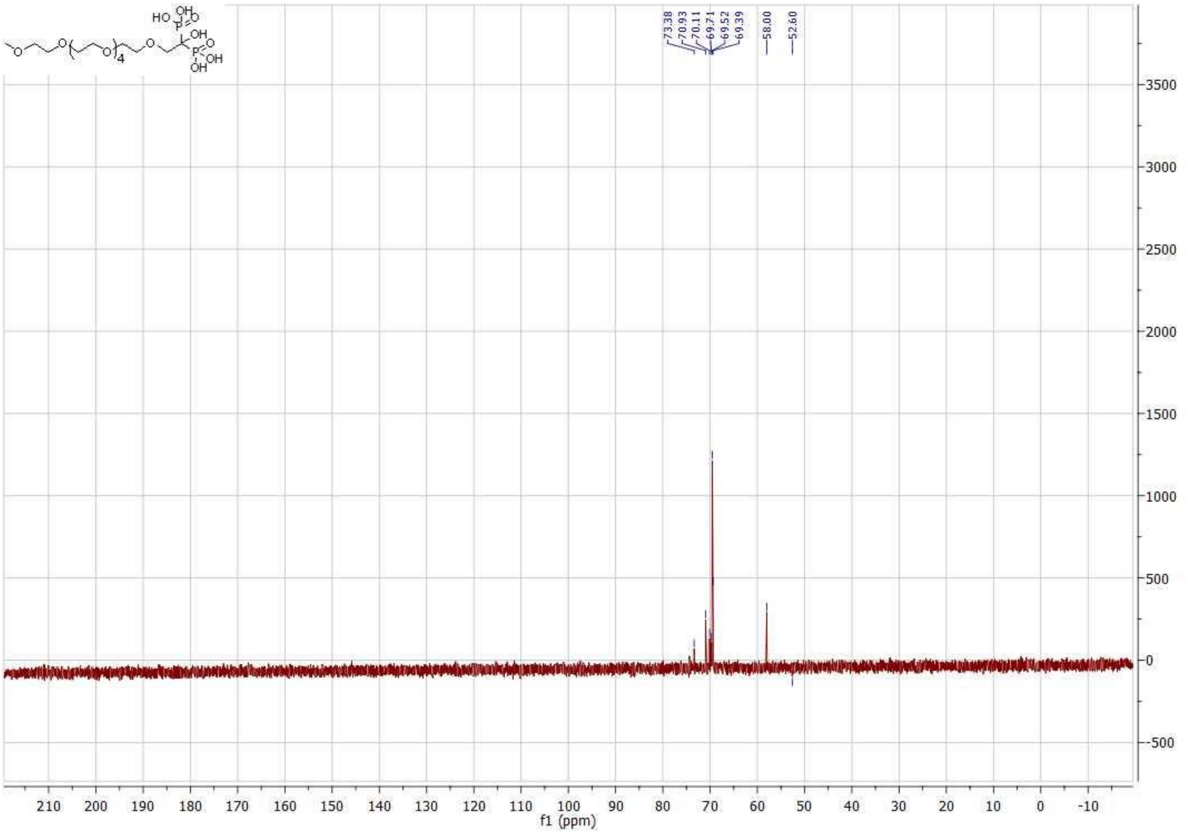

^{13}C NMR spectrum of compound **1’a** in D_2O

(3,6,9,12,15,18,21,24,27,30,33,36-Dodecaoxa-1-hydroxy-heptatriacosane-1,1 diyl)bisphosphonic acid (**1’b**)

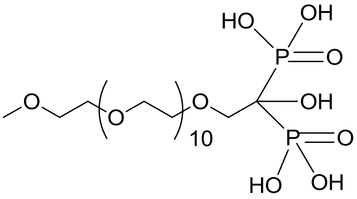

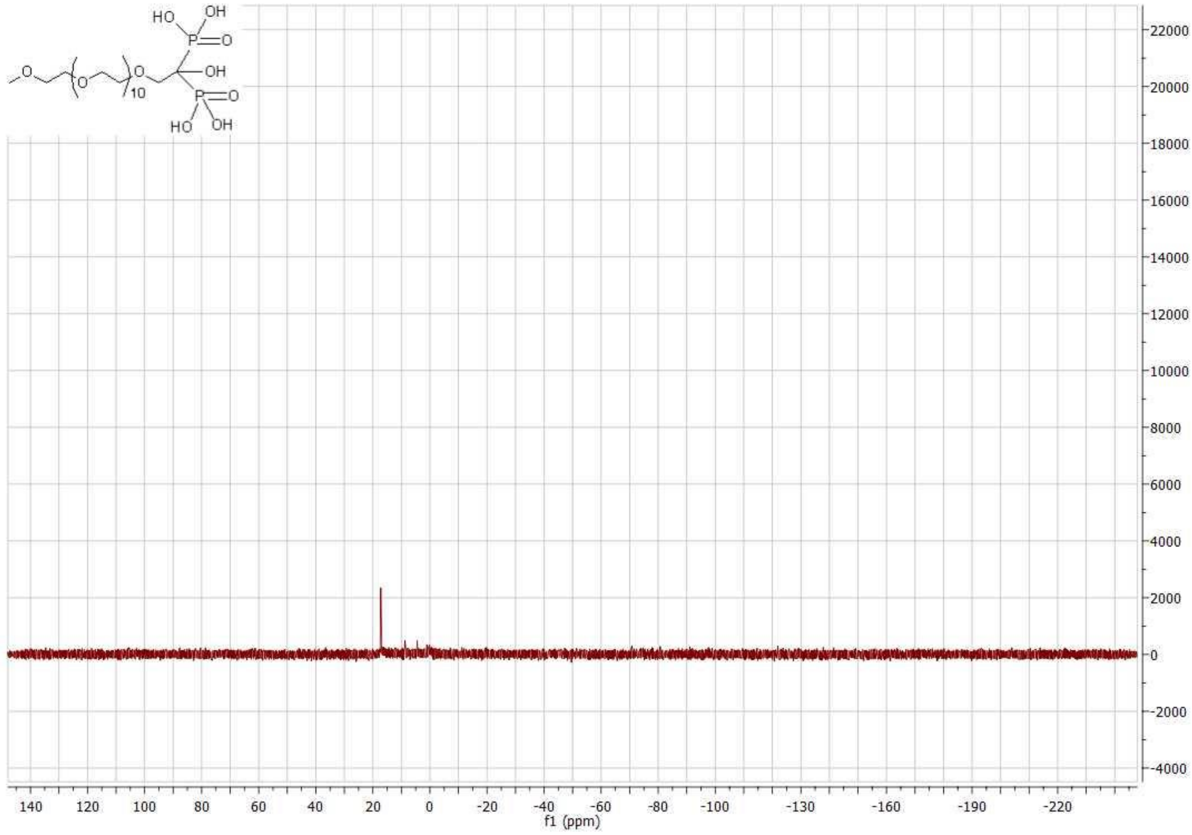

^{31}P NMR spectrum of compound **1’b** in D_2O

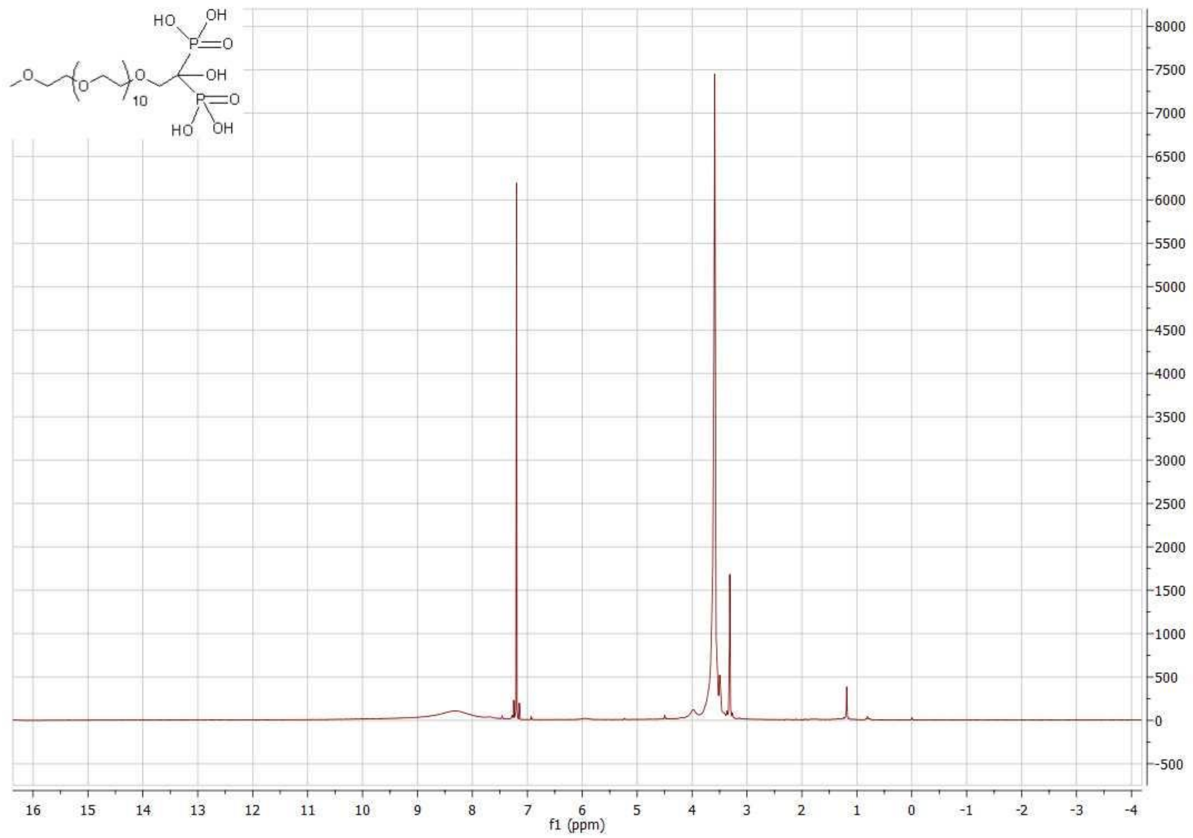

^{1}H NMR spectrum of compound **1’b** in D_2O

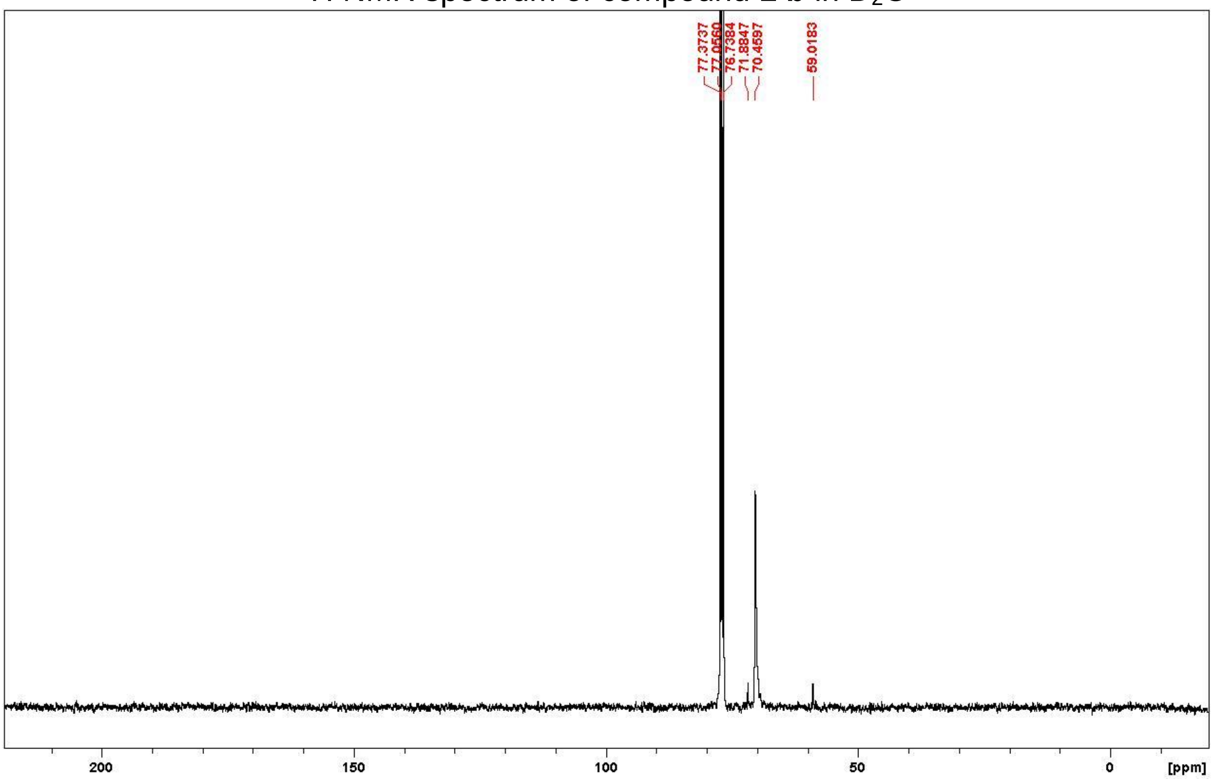

^{13}C NMR spectrum of compound **1’b** in D_2O

(14-Azido-1-hydroxy-3,6,9,12-tetraoxatetradecane-1,1-diyl)bisphosphonic acid (**16a**).

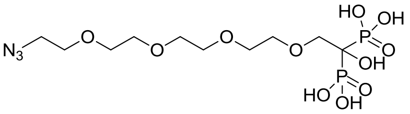

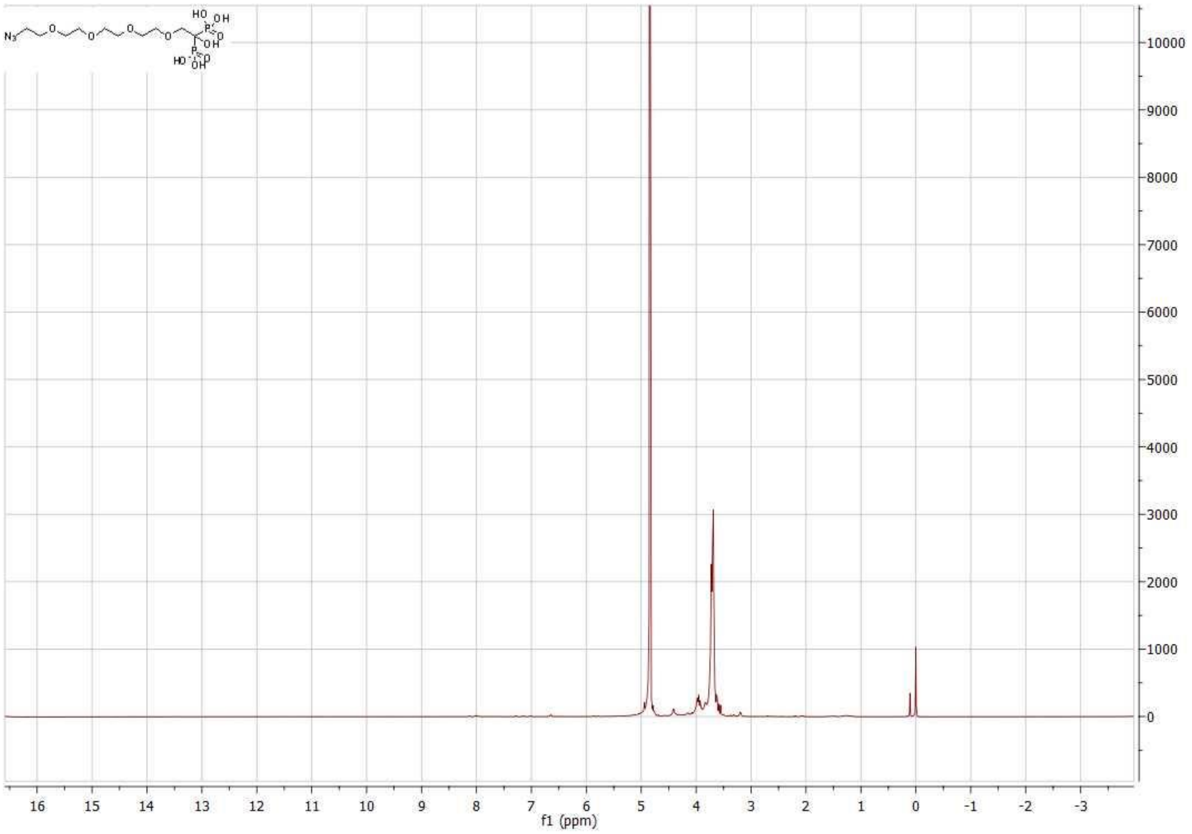

^{1}H NMR spectrum of compound **16a** in D_2O

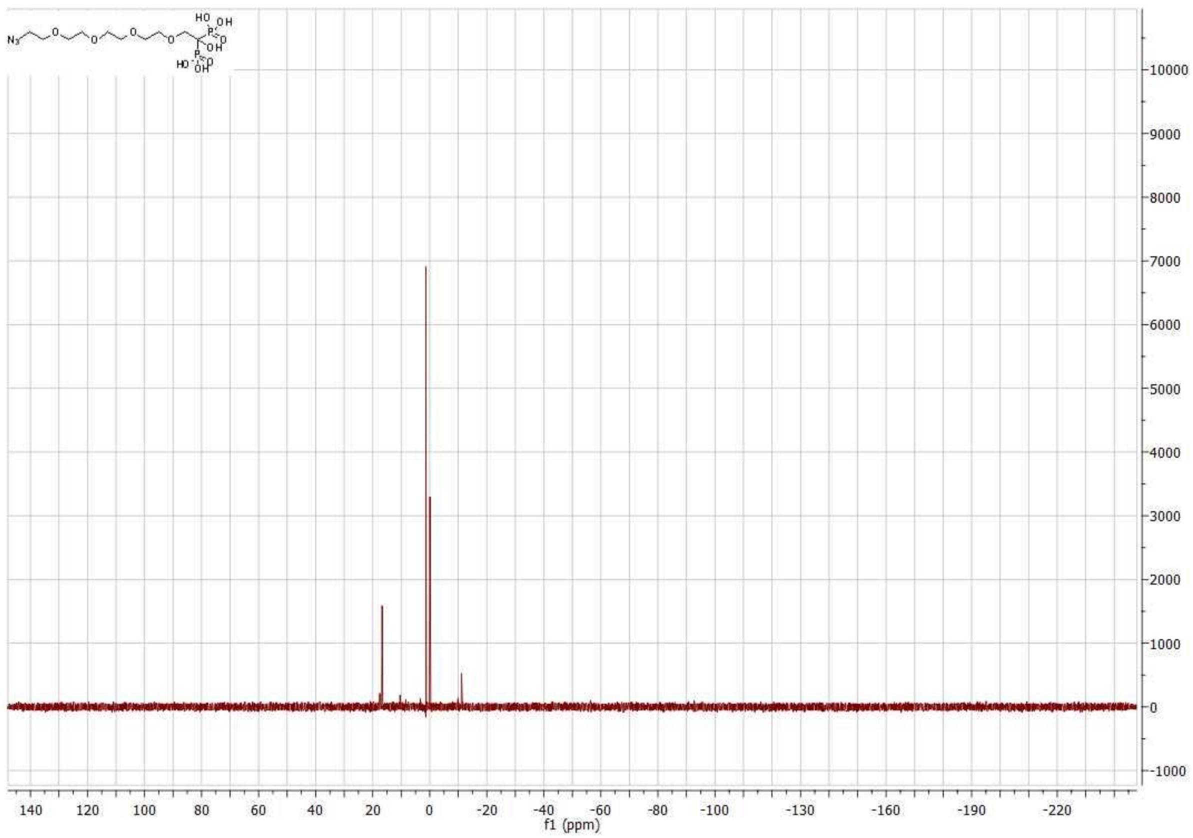

^{31}P NMR spectrum of compound **16a** in D_2O

(20-Azido-1-hydroxy-3,6,9,12,15,18-hexaoxaicosane-1,1-diyl)bisphosphonic acid (**16b**).

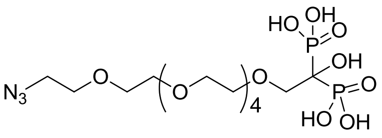

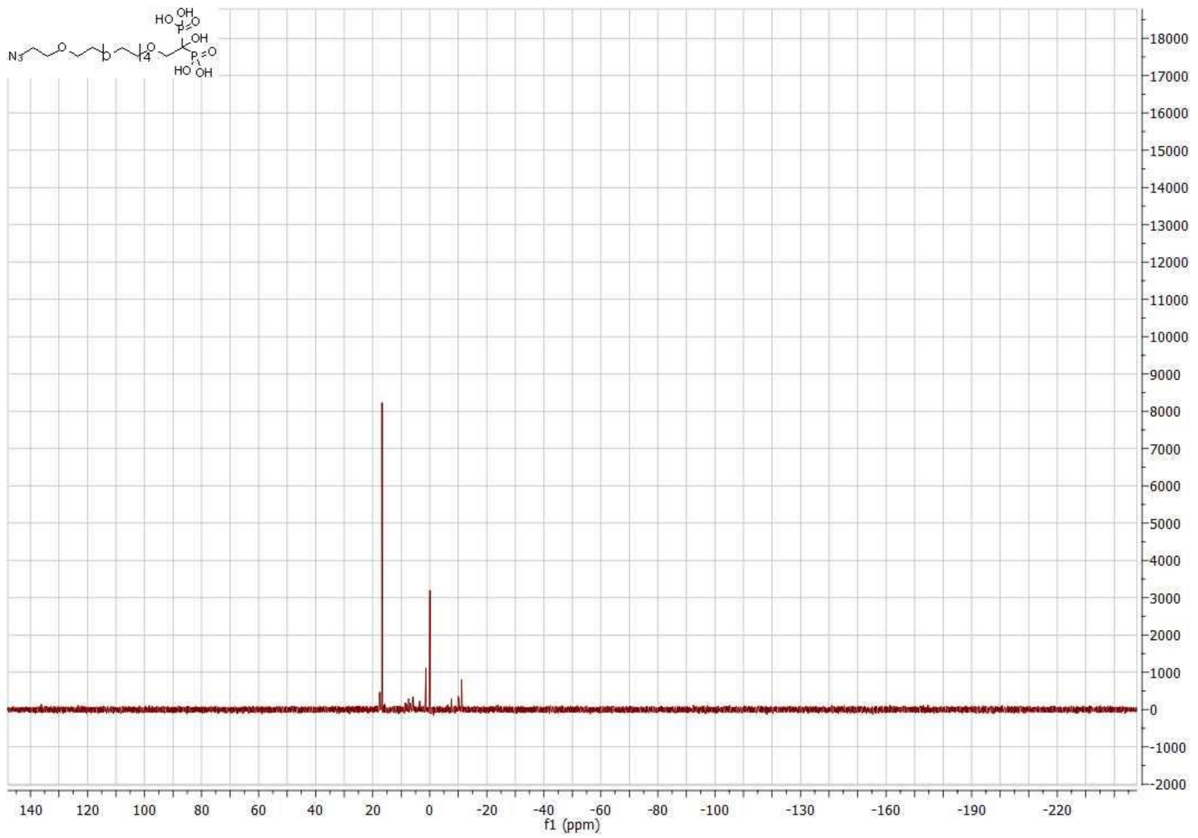

^{31}P NMR spectrum of compound **16b** in D_2O

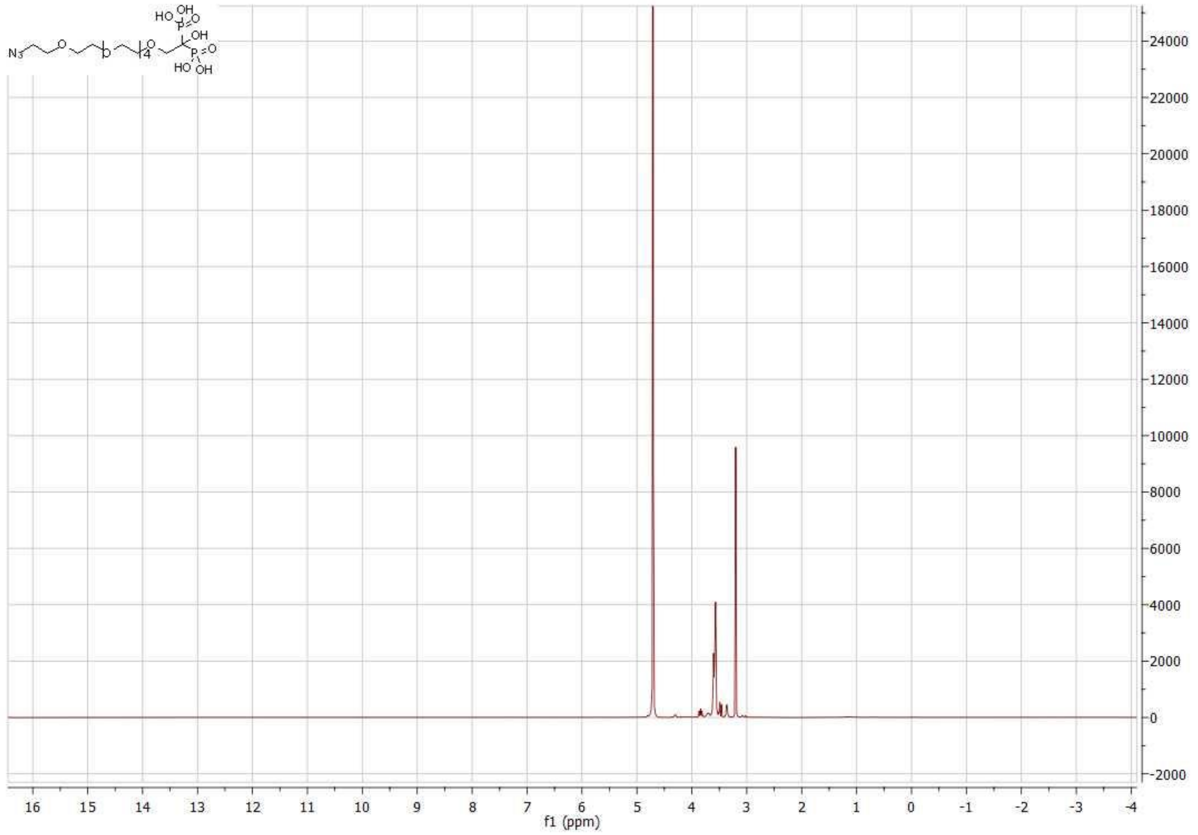

^{1}H NMR spectrum of compound **16a** in D_2O

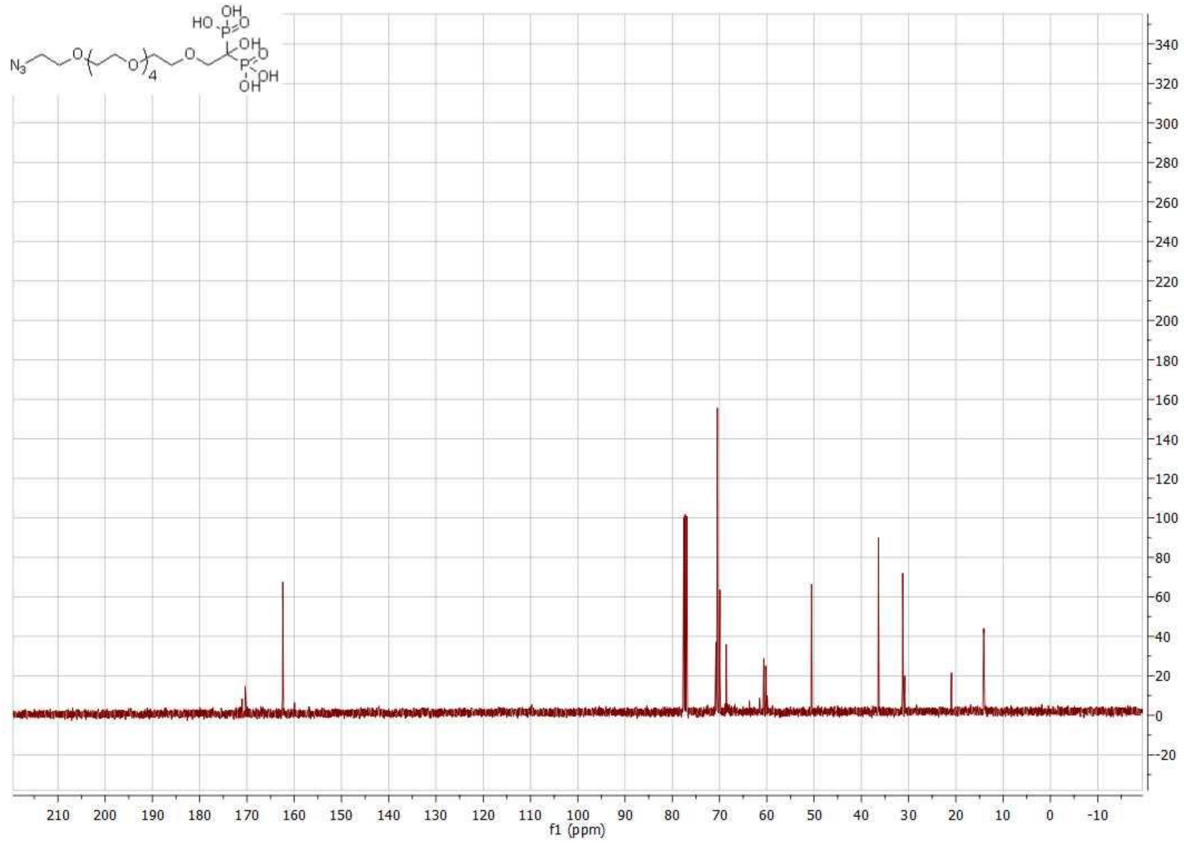

^{13}C NMR spectrum of compound **16b** in D_2O

(1-Hydroxy-14-oxo-3,6,9,12,15-pentaoxaheptadecane-1,1-diyl)bisphosphonic acid (**22**)

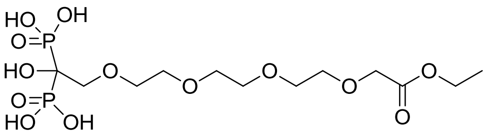

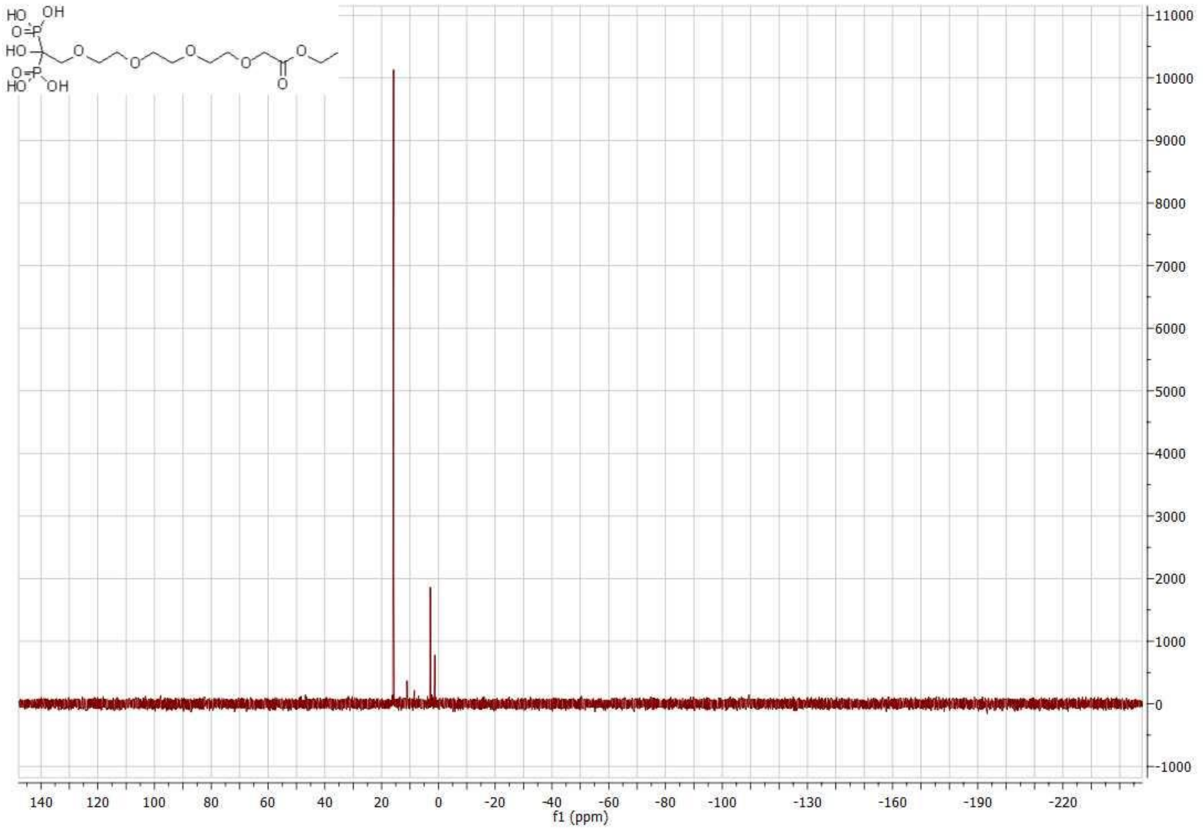

^{31}P NMR spectrum of compound **22** in D_2O

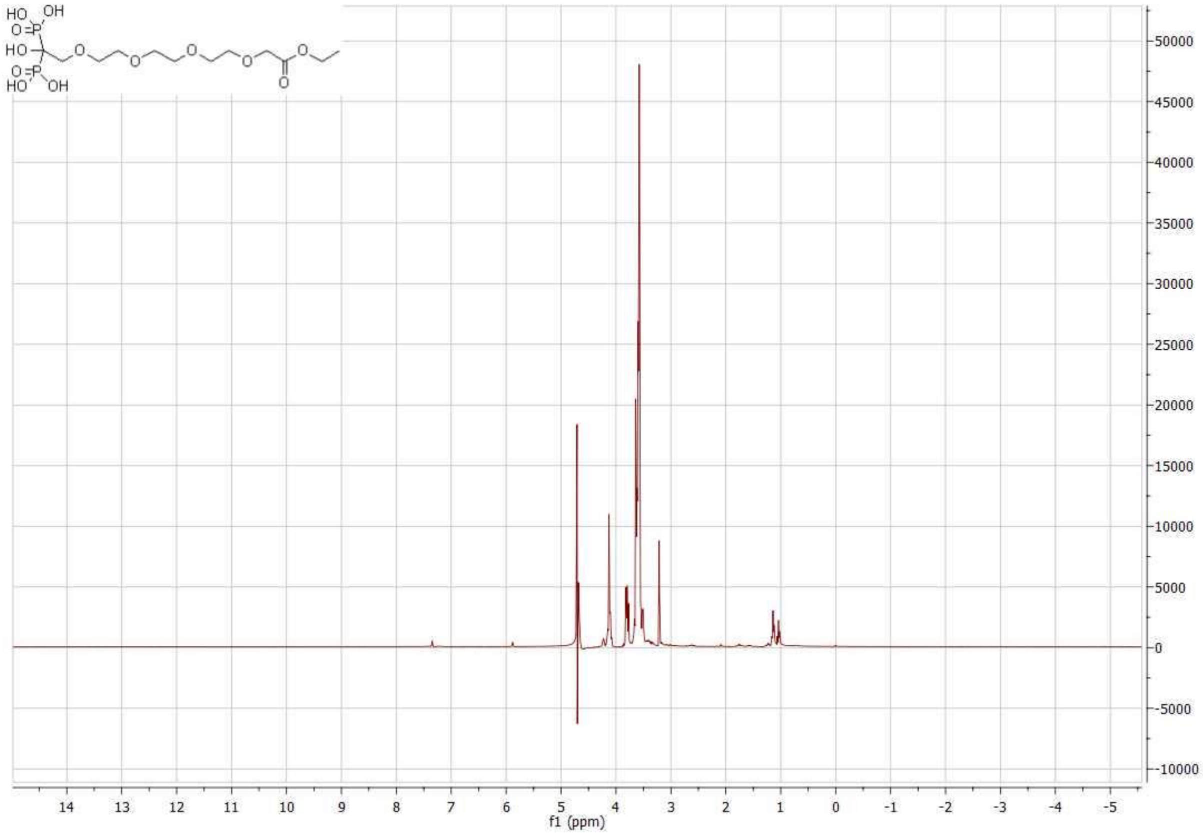

^{1}H NMR spectrum of compound **22** in D_2O

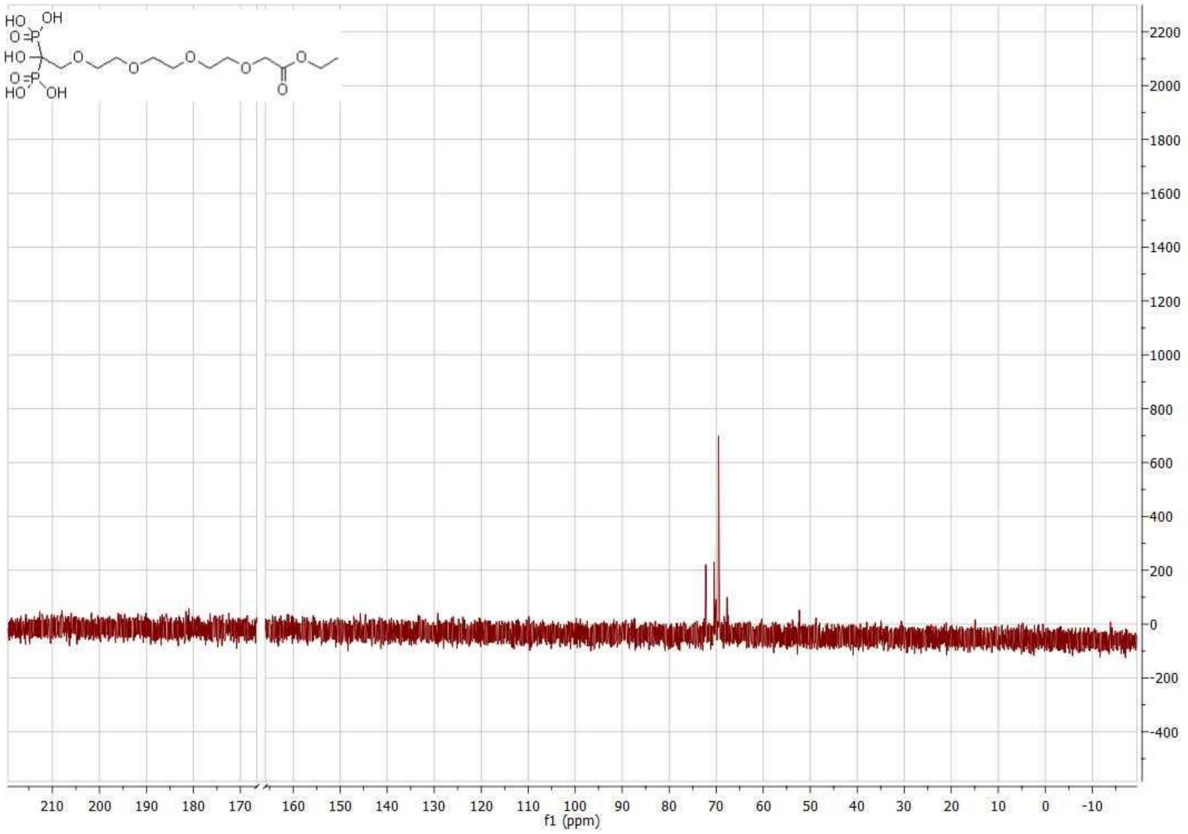

^{13}C NMR spectrum of compound **22** in D_2O

1-Phenyl-2,5,8,11-tetraoxatridecan-13-al (**4**).

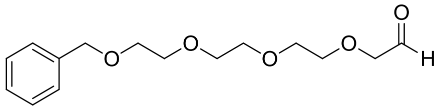

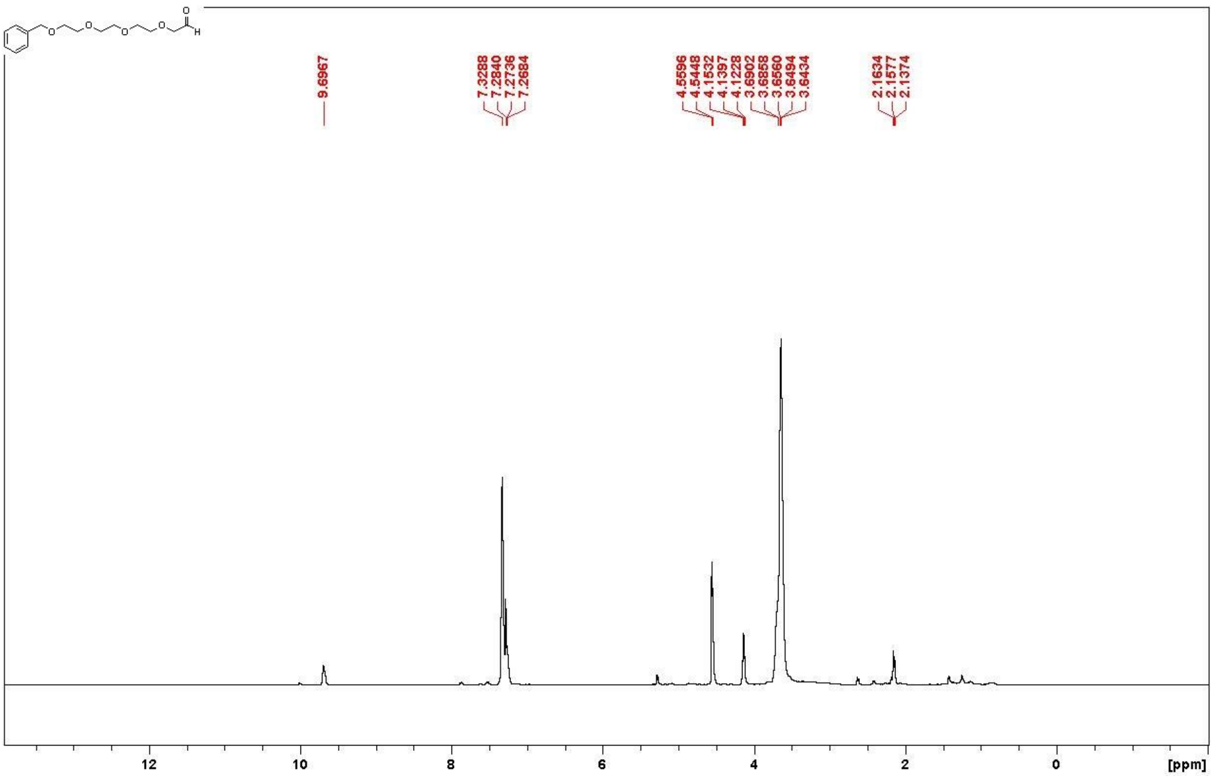

^{1}H NMR spectrum of compound **4** in $CDCl_3$

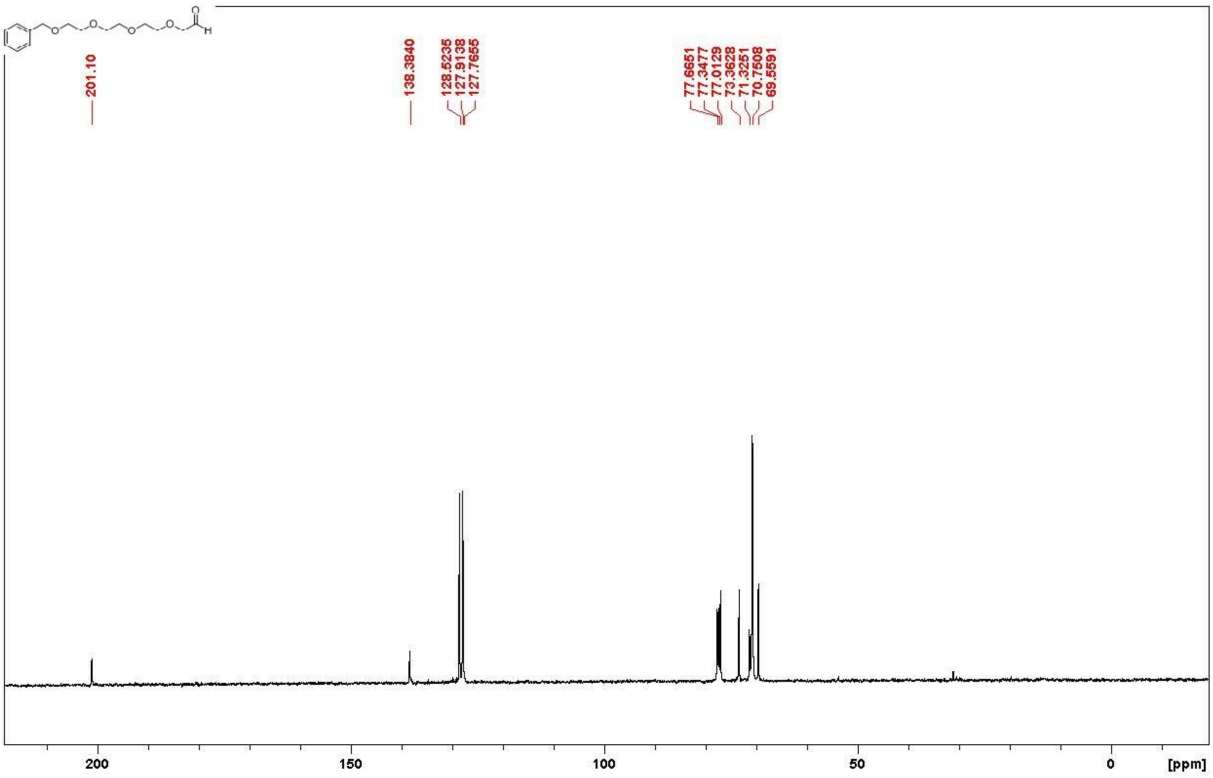

^{13}C NMR spectrum of compound **4** in $CDCl_3$

2,5,8,11,14,17,20-Heptaoxadocosan-22-al (**5a**).

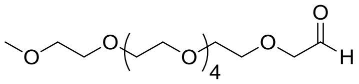

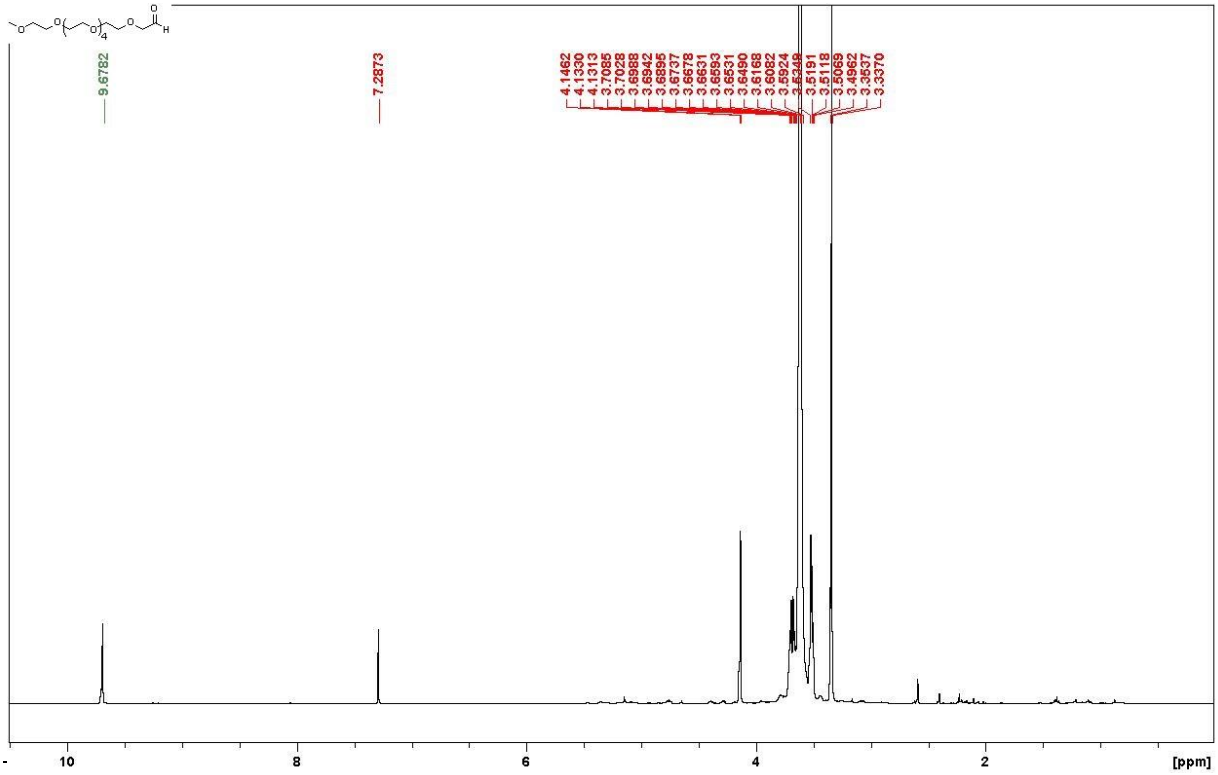

^{1}H NMR spectrum of compound **5a** in $CDCl_3$

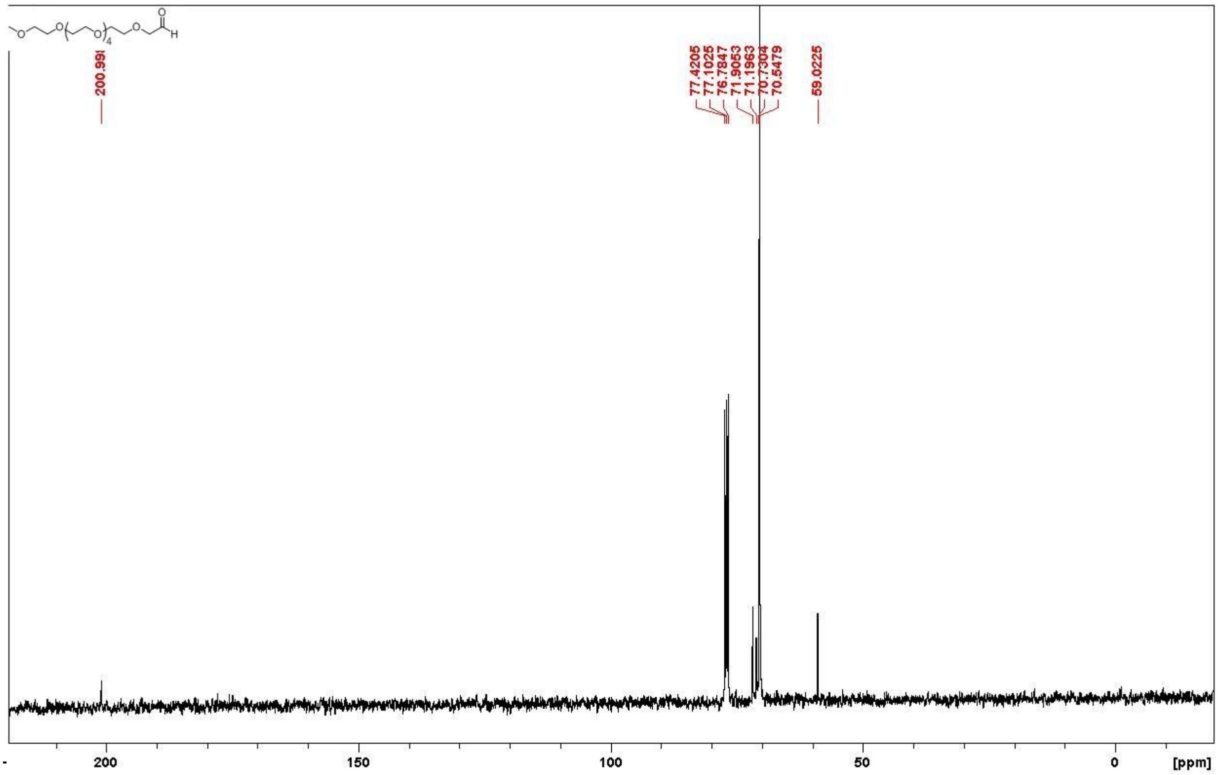

^{13}C NMR spectrum of compound **5a** in $CDCl_3$

2,5,8,11,14,17,20,23,26,29,32,35-Dodecaoxaheptatria-contan-37-al (**5b**).

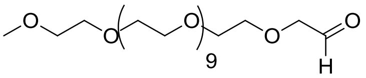

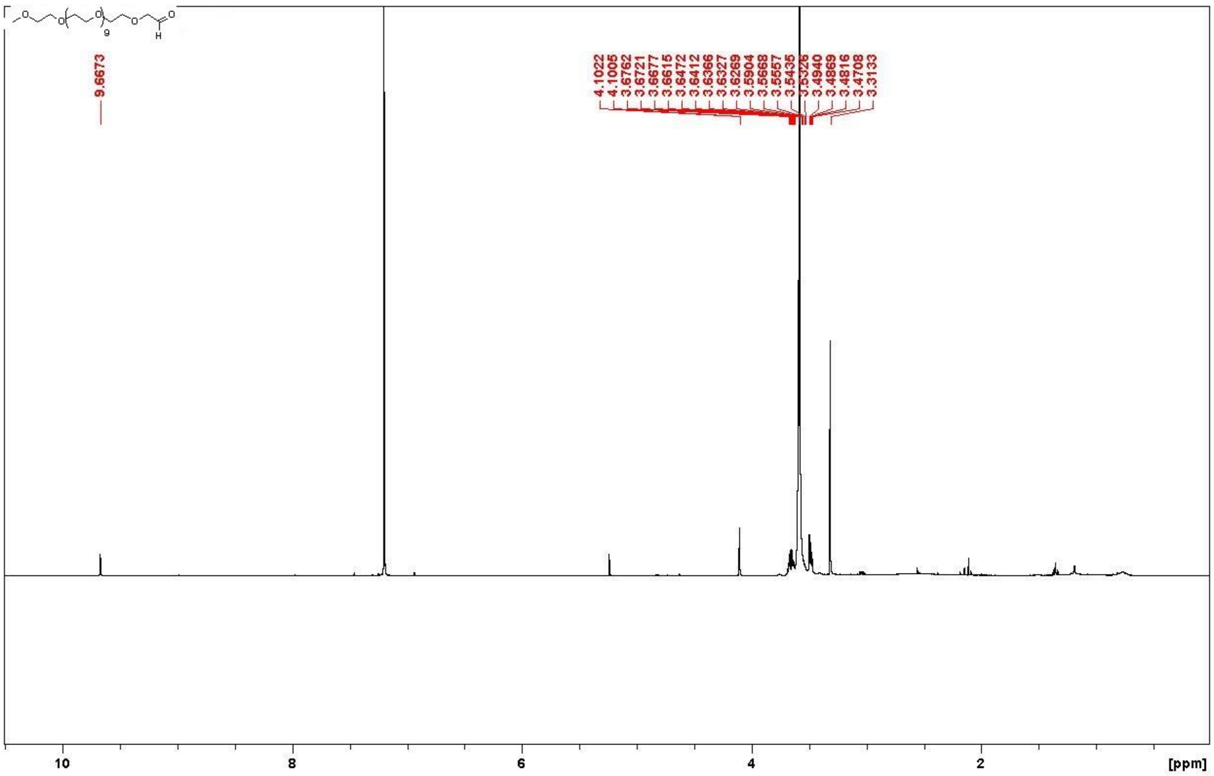

^{1}H NMR spectrum of compound **5b** in $CDCl_3$

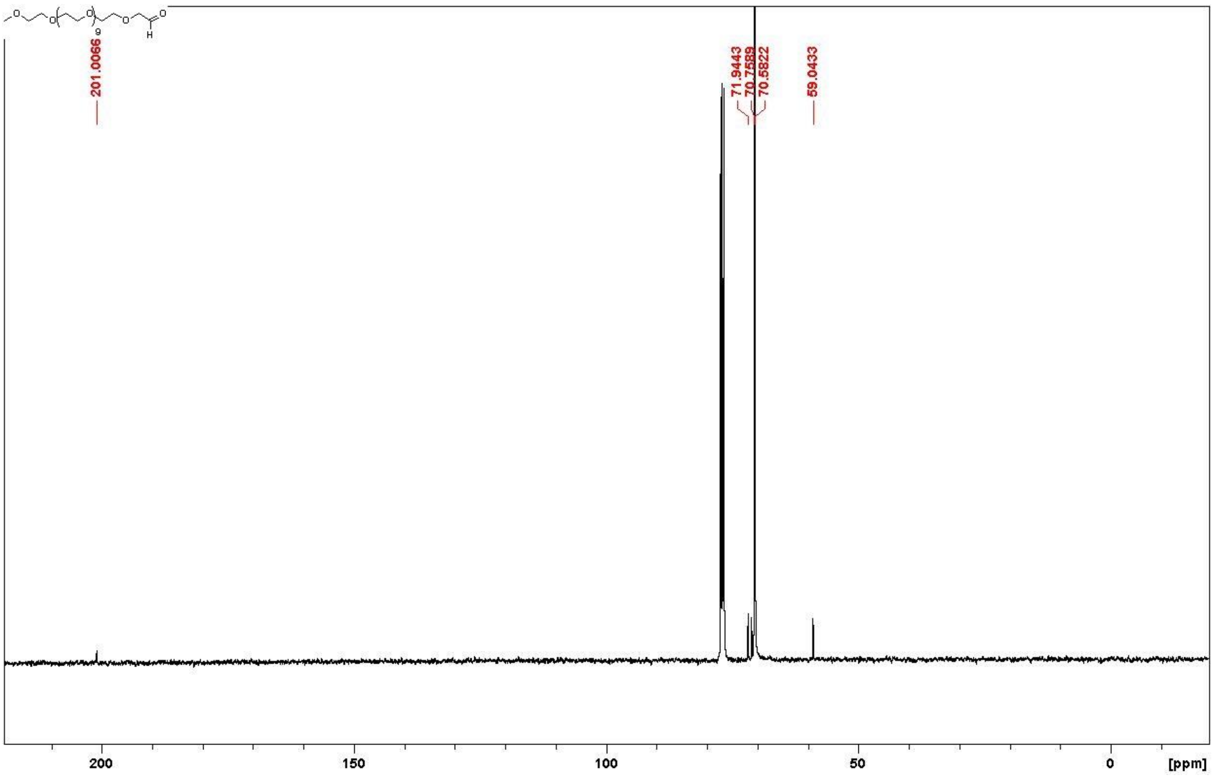

^{13}C NMR spectrum of compound **5b** in $CDCl_3$

Ethyl 14-oxo-3,6,9,12-tetraoxatetradecan-1-oate (**19**)

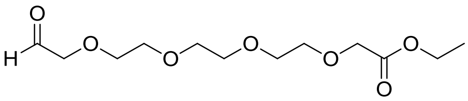

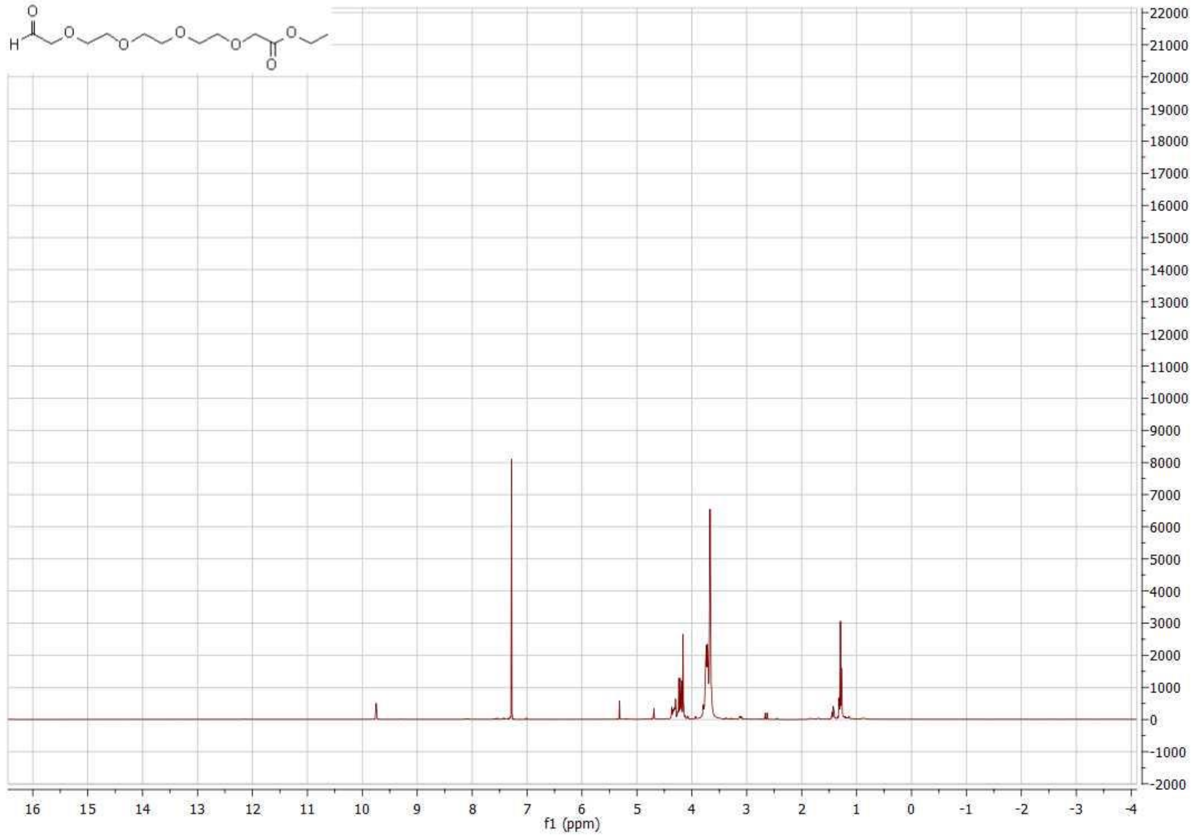

^{1}H NMR spectrum of compound **19** in $CDCl_3$

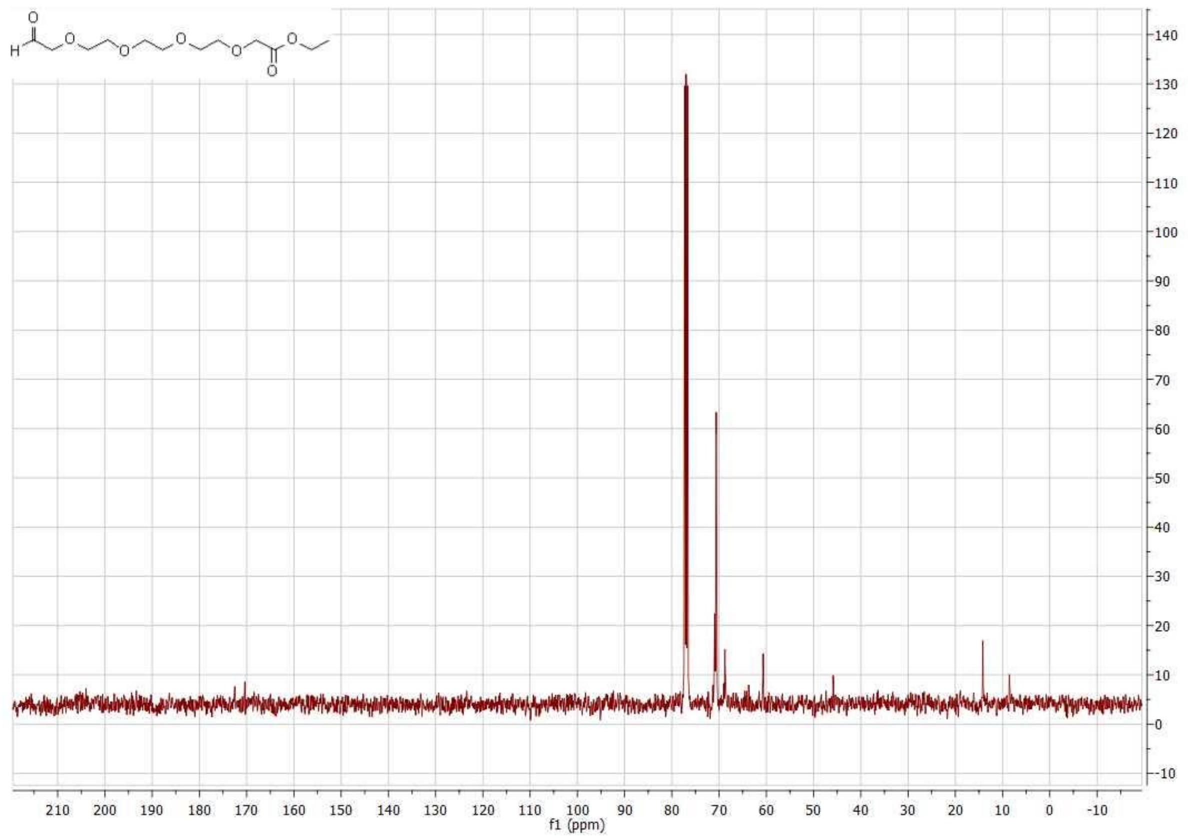

^{13}C NMR spectrum of compound **19** in $CDCl_3$

2,5,8,11,14,17,20,23,26,29,32,35-Dodecaoxaheptatriacontan-37-oic acid (**7b**).

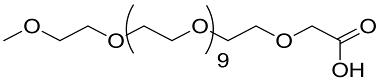

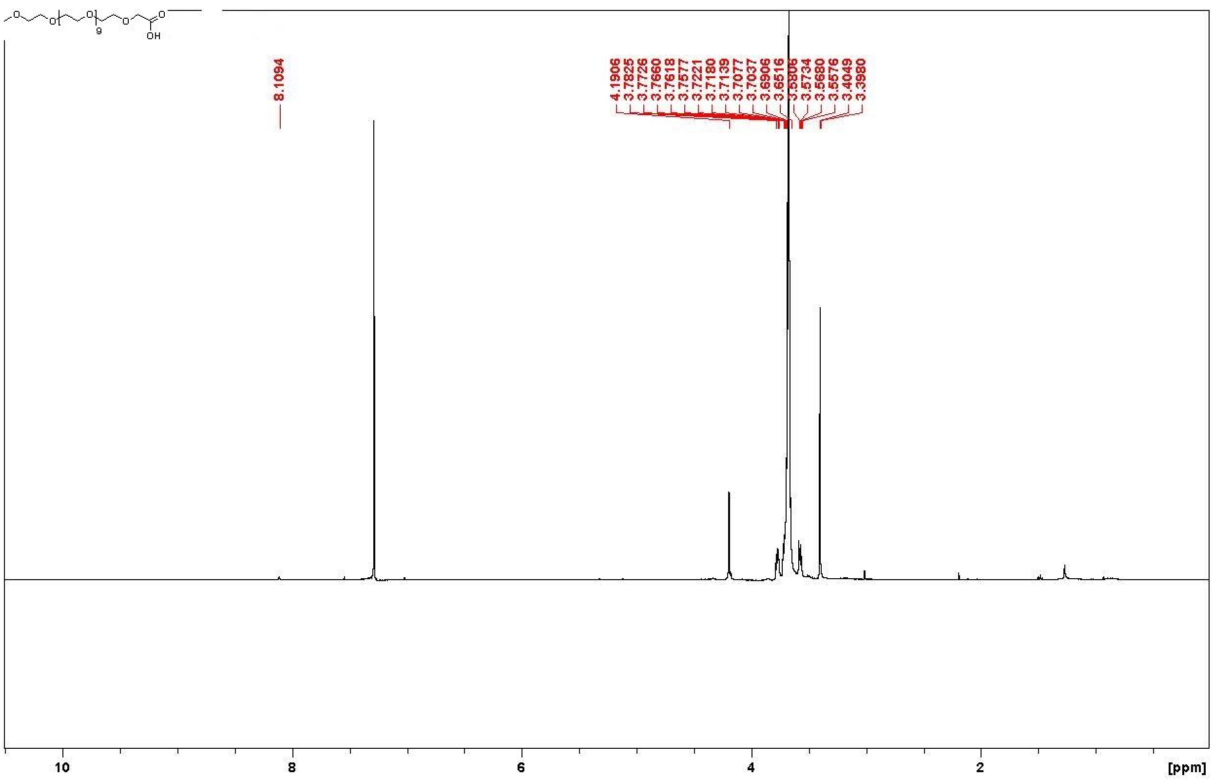

^{1}H NMR spectrum of compound **7b** in $CDCl_3$

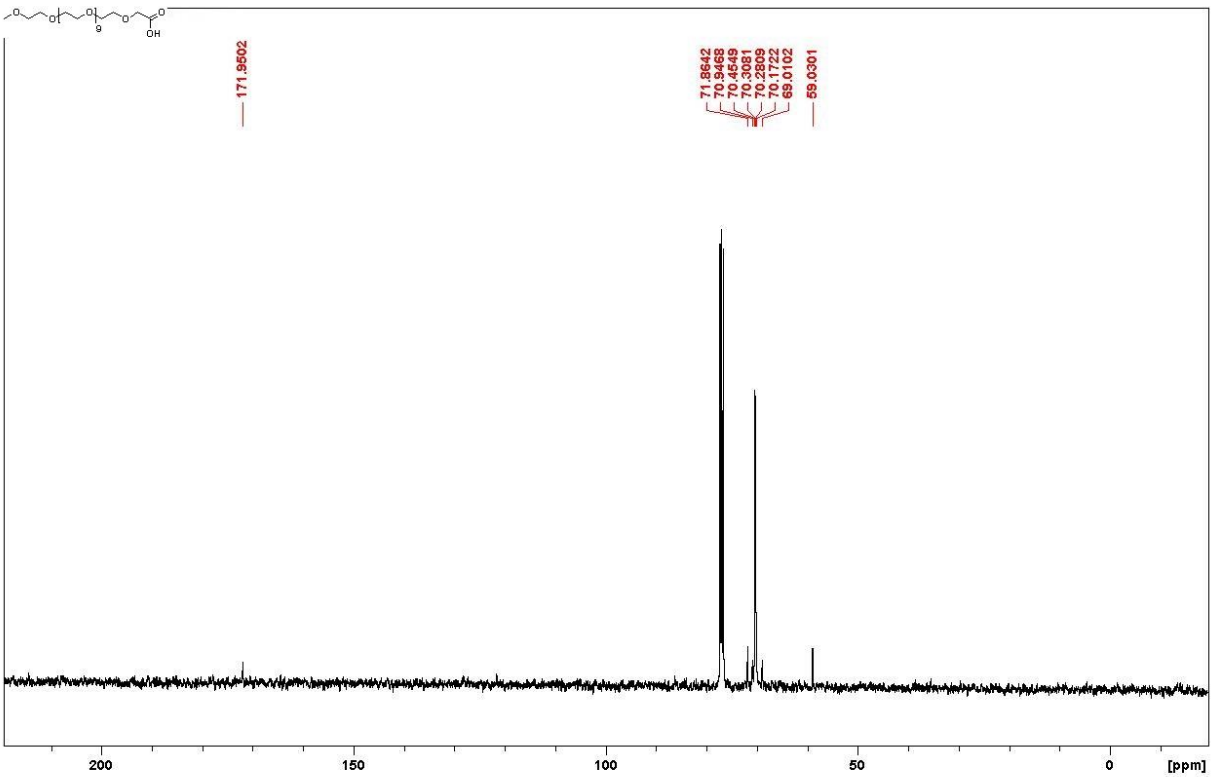

^{13}C NMR spectrum of compound **7b** in $CDCl_3$

14-Oxo-3,6,9,12,15-pentaoxaheptadecan-1-oic acid (**20**).

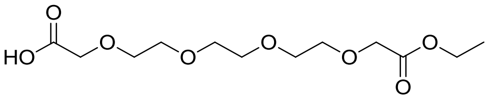

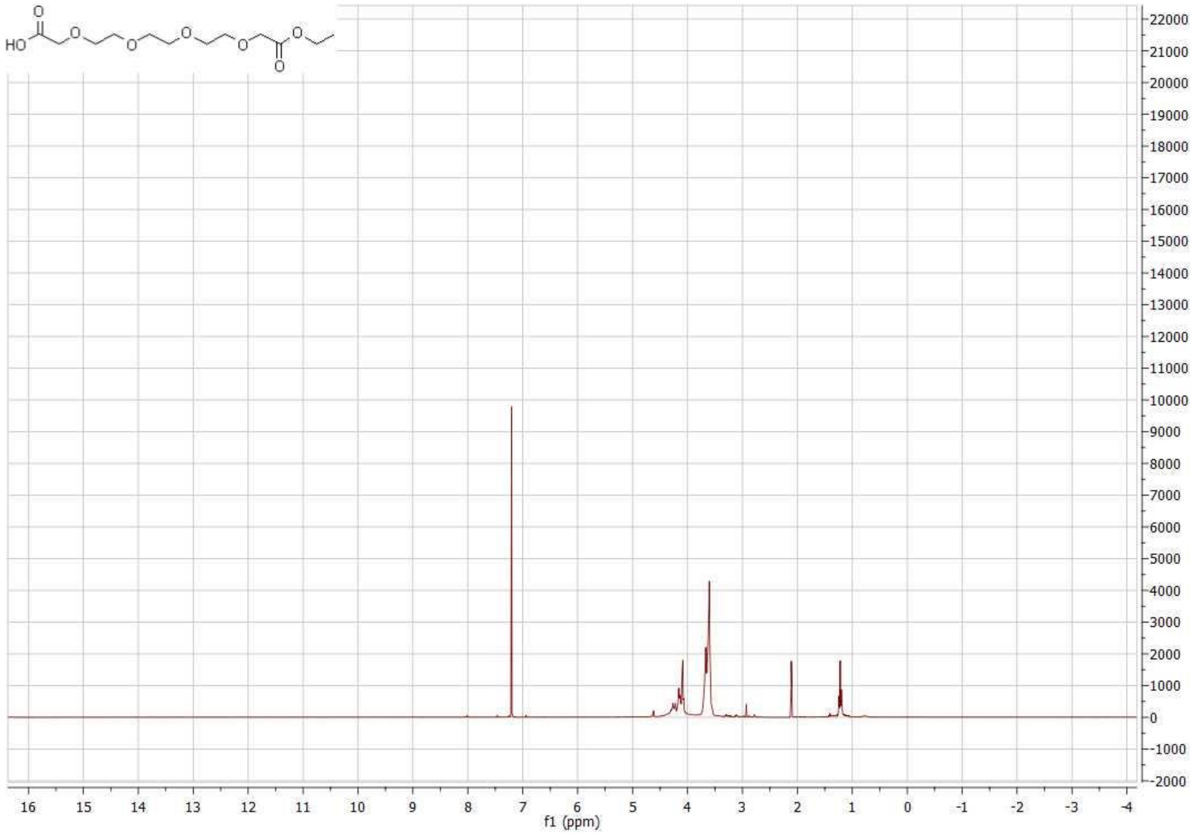

^{1}H NMR spectrum of compound **20** in $CDCl_3$

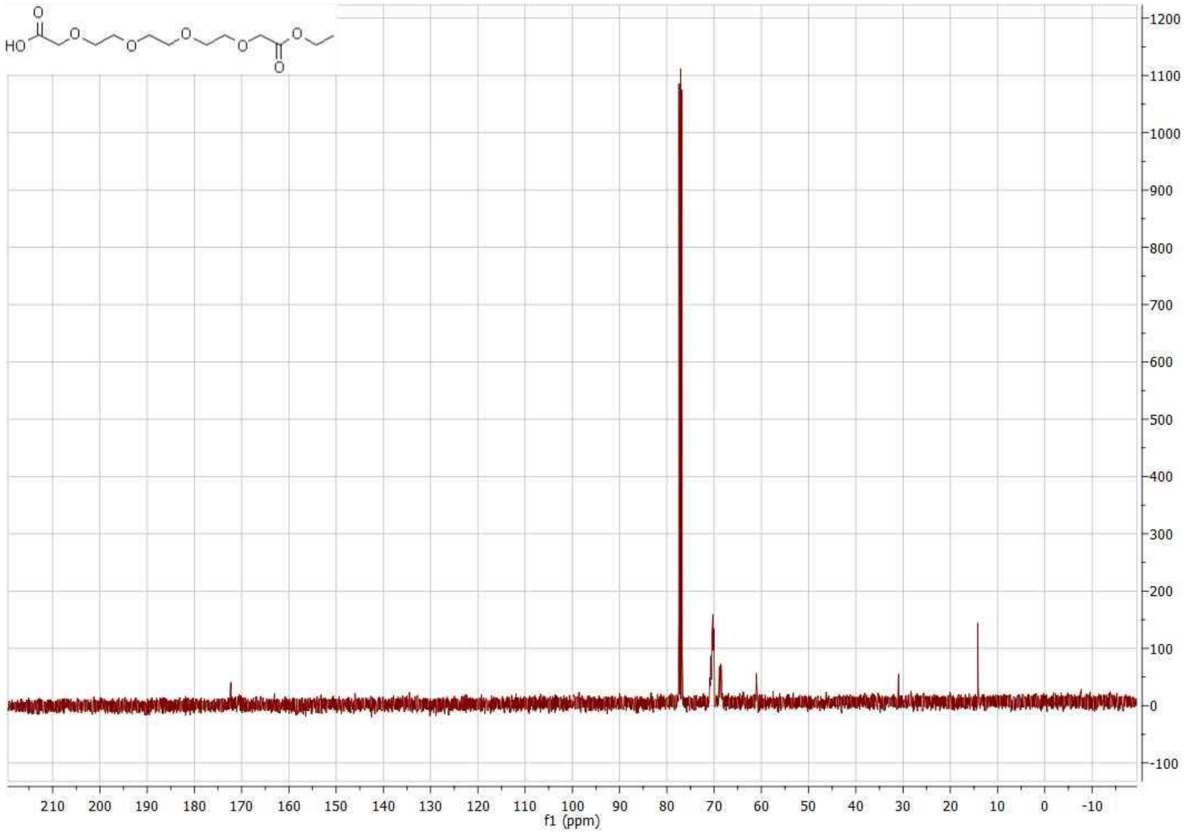

^{13}C NMR spectrum of compound **20** in $CDCl_3$

(14-Ammonio-1-hydroxy-3,6,9,12-tetraoxatetradecane-1,1-diyl)bis(hydrogen phosphonate) monosodium salt (**17a**).

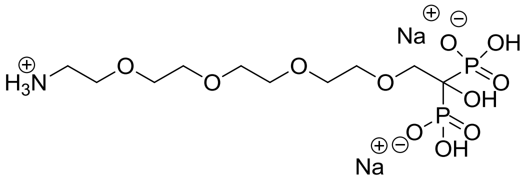

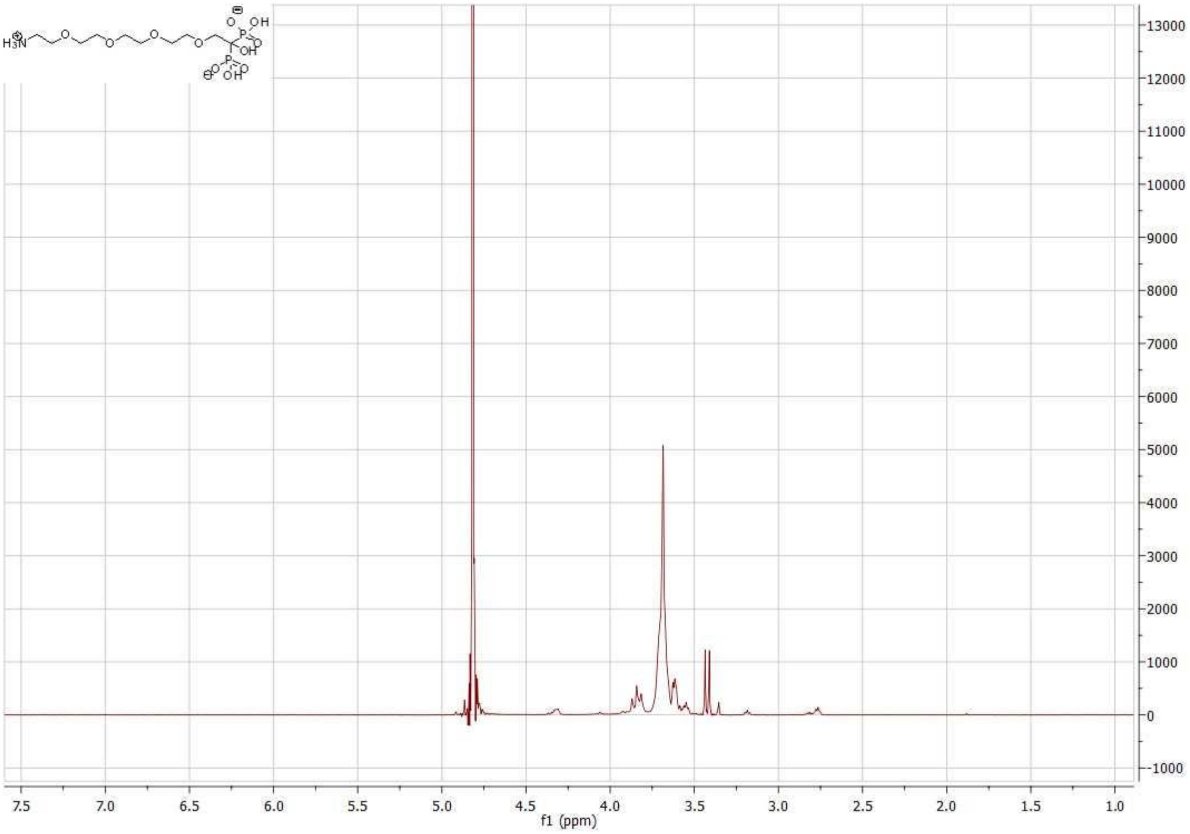

^{1}H NMR spectrum of compound **17a** in D_2O

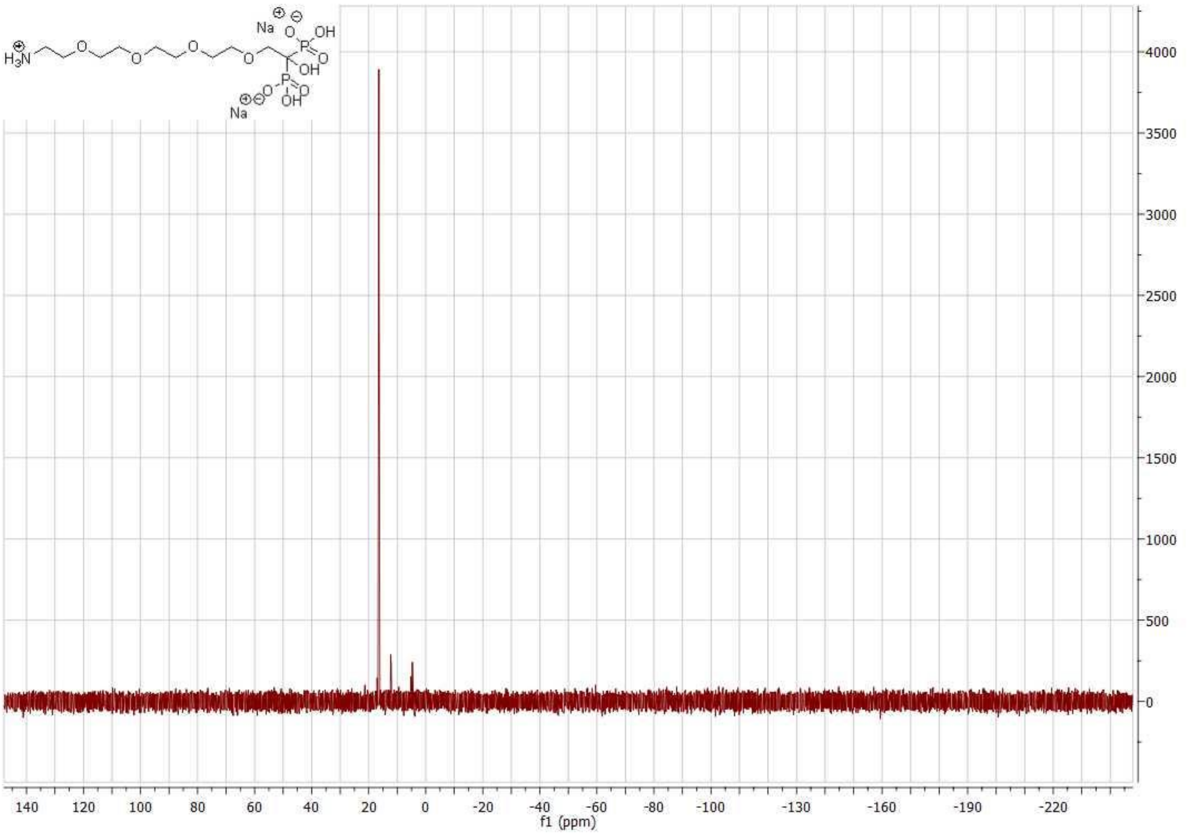

^{31}P NMR spectrum of compound **17a** in D_2O

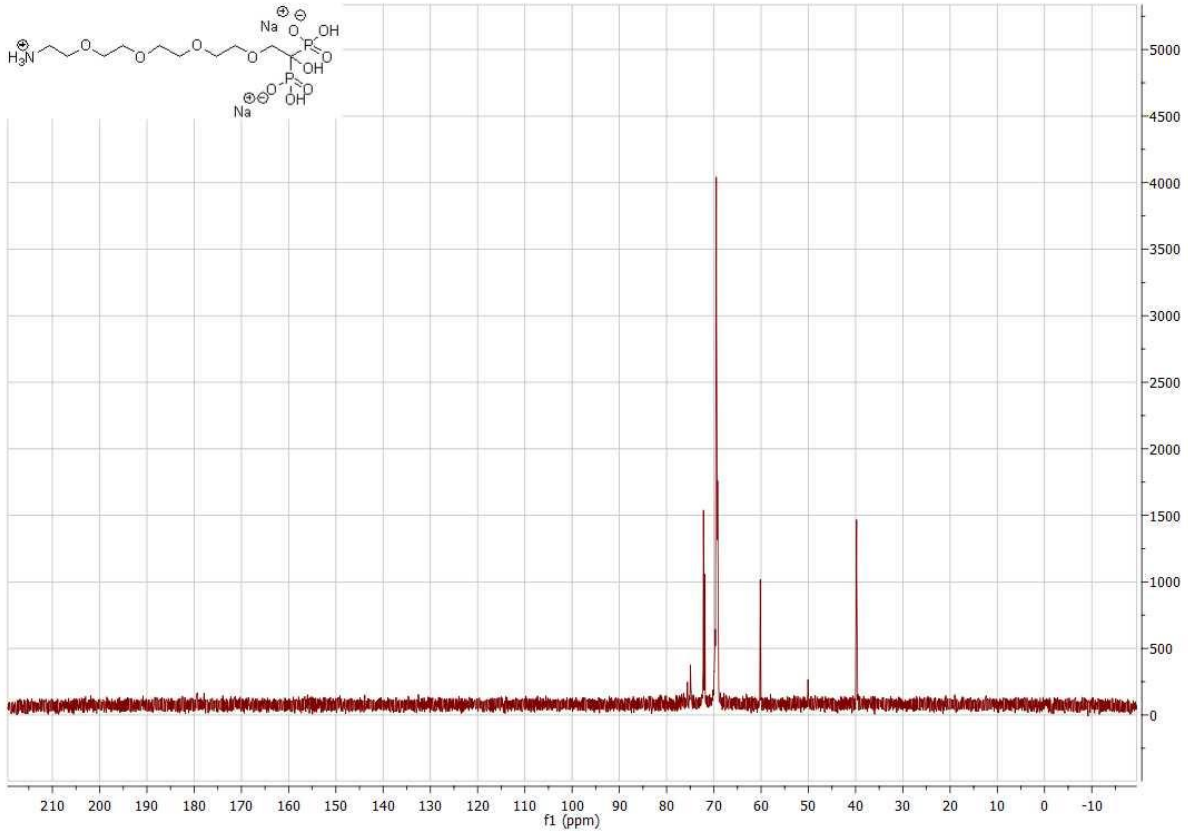

^{13}C NMR spectrum of compound **17a** in D_2O

(20-Ammonio-1-hydroxy-3,6,9,12,15,18-hexaoxaicosane-1,1-diyl)bis(hydrogen phosphonate) monosodium salt (**17b**)

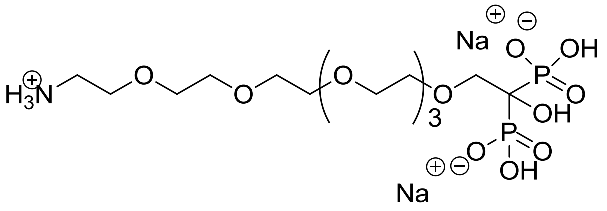

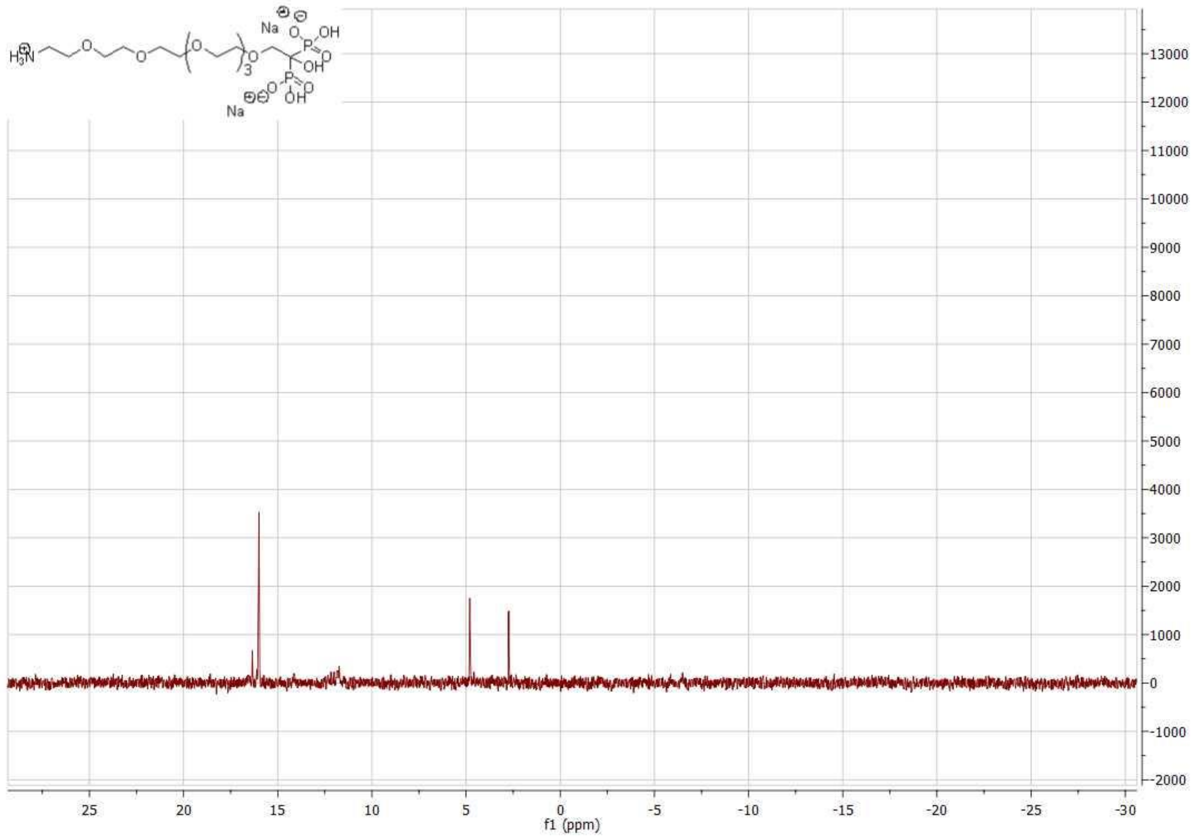

^{31}P NMR spectrum of compound **17b** in D_2O

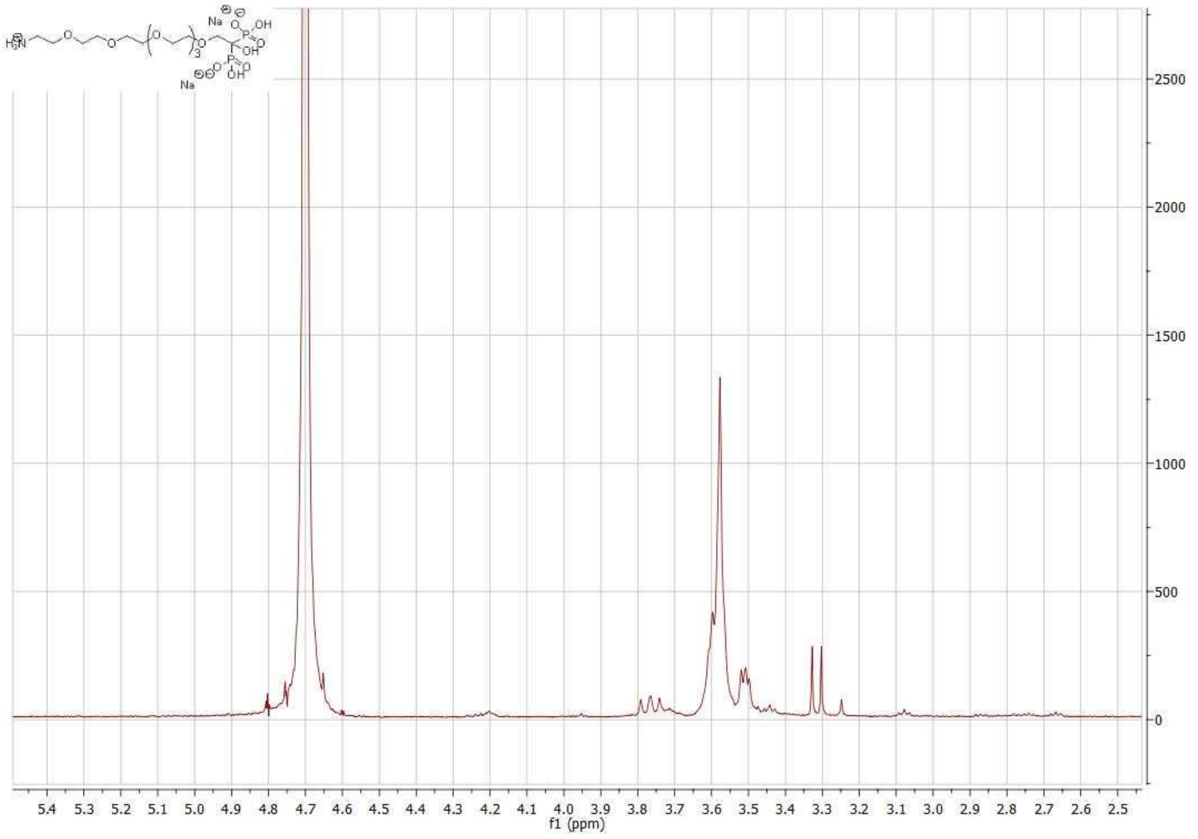

^{1}H NMR spectrum of compound **17b** in D_2O

Ethyl 20-azido-3,6,9,12,15,18-hexaoxaicosan-1-oate (**13b**)

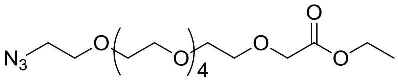

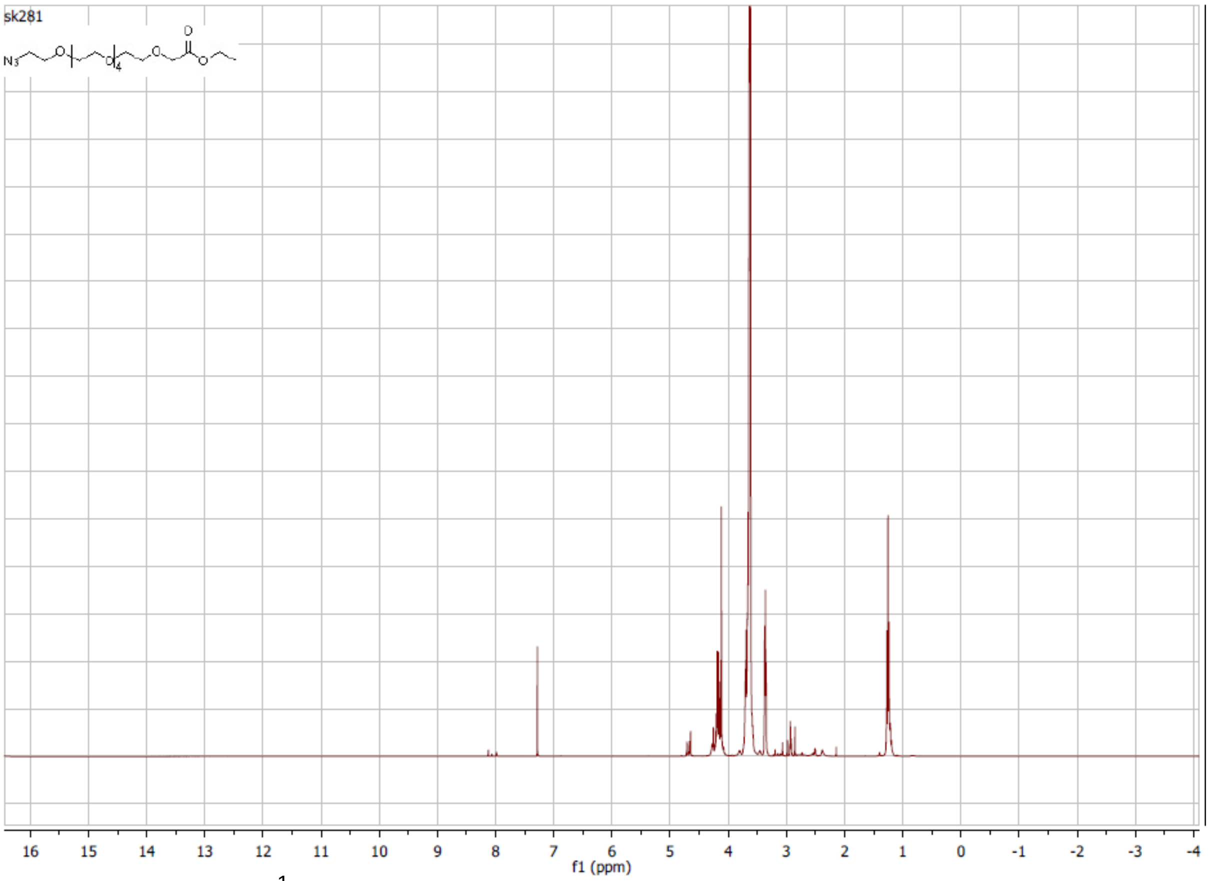

^{1}H NMR spectrum of compound **13b** in $CDCl_3$

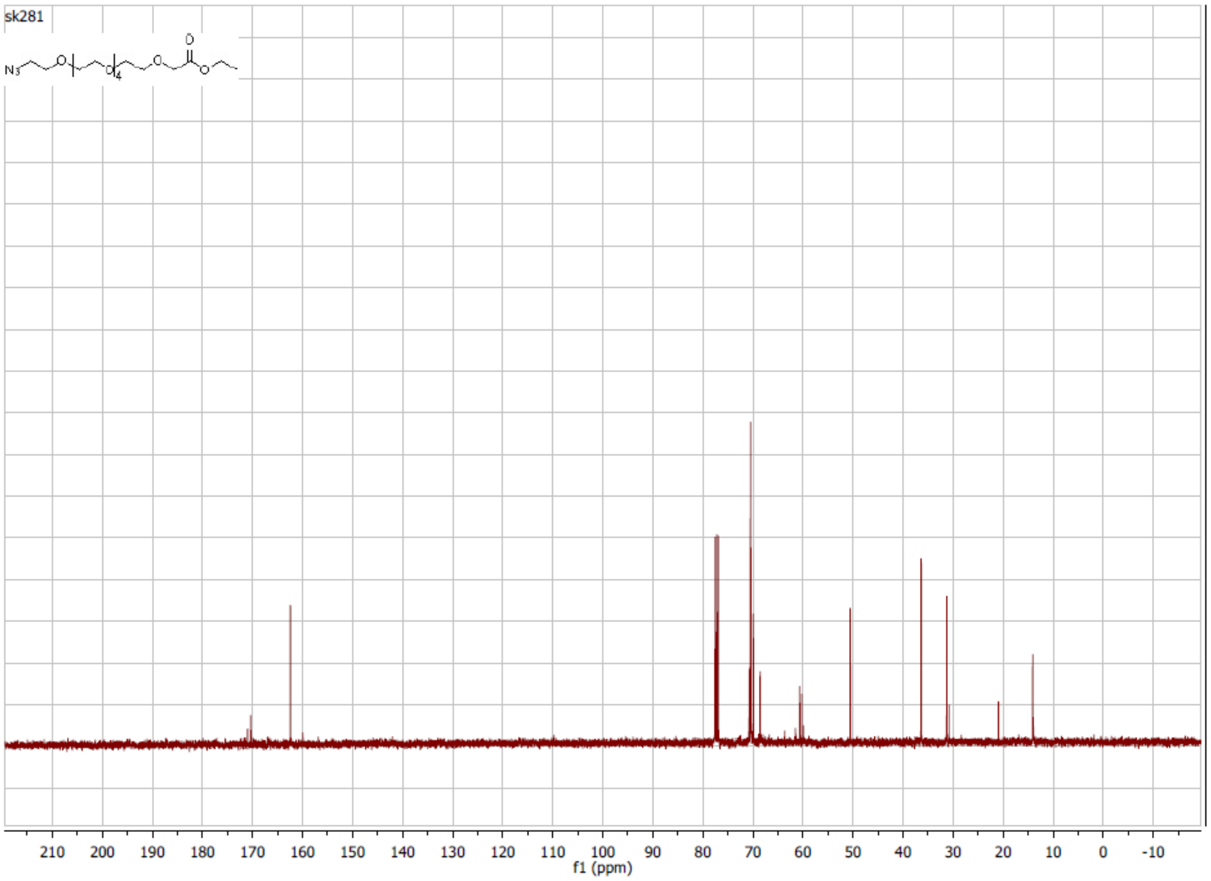

^{13}C NMR spectrum of compound **13b** in $CDCl_3$

14-Hydroxy-14,14-diphosphono-3,6,9,12-tetraoxatetradecan-1-oic acid (**23**)

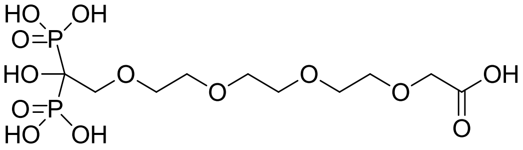

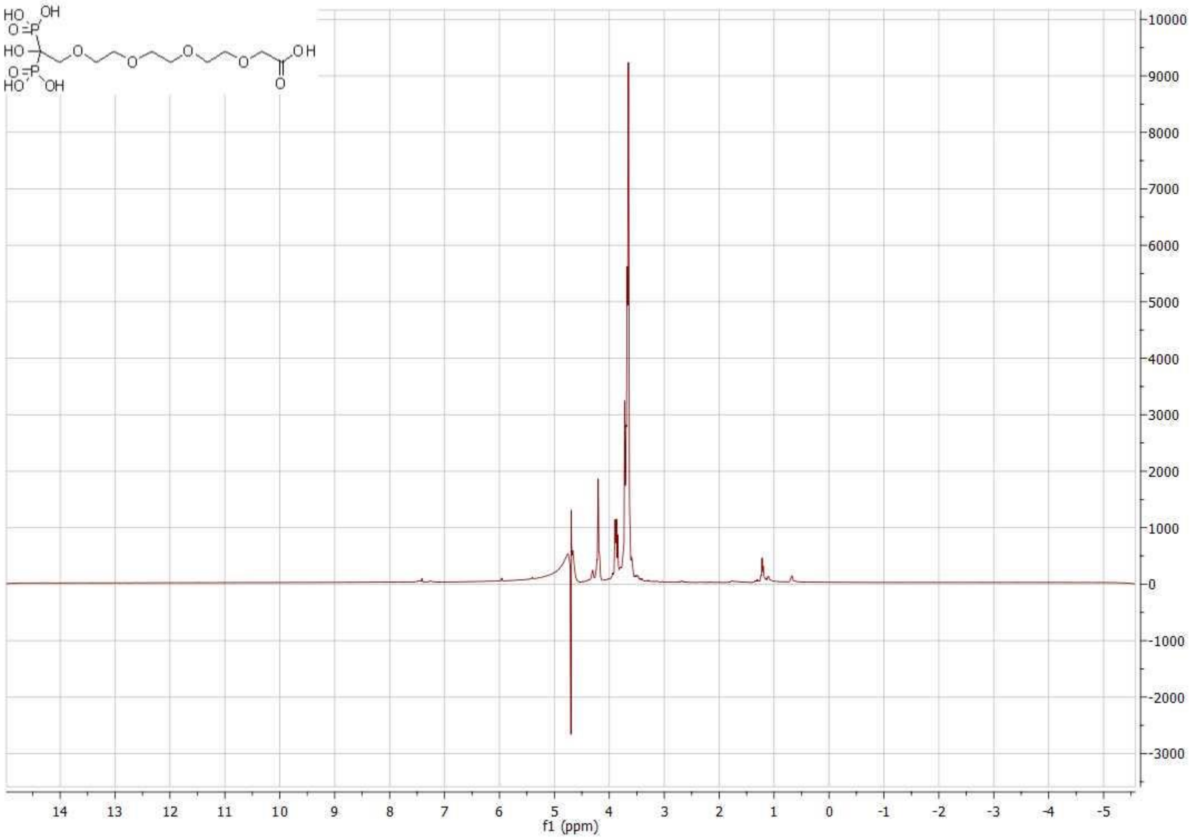

^{1}H NMR spectrum of compound **23** in D_2O

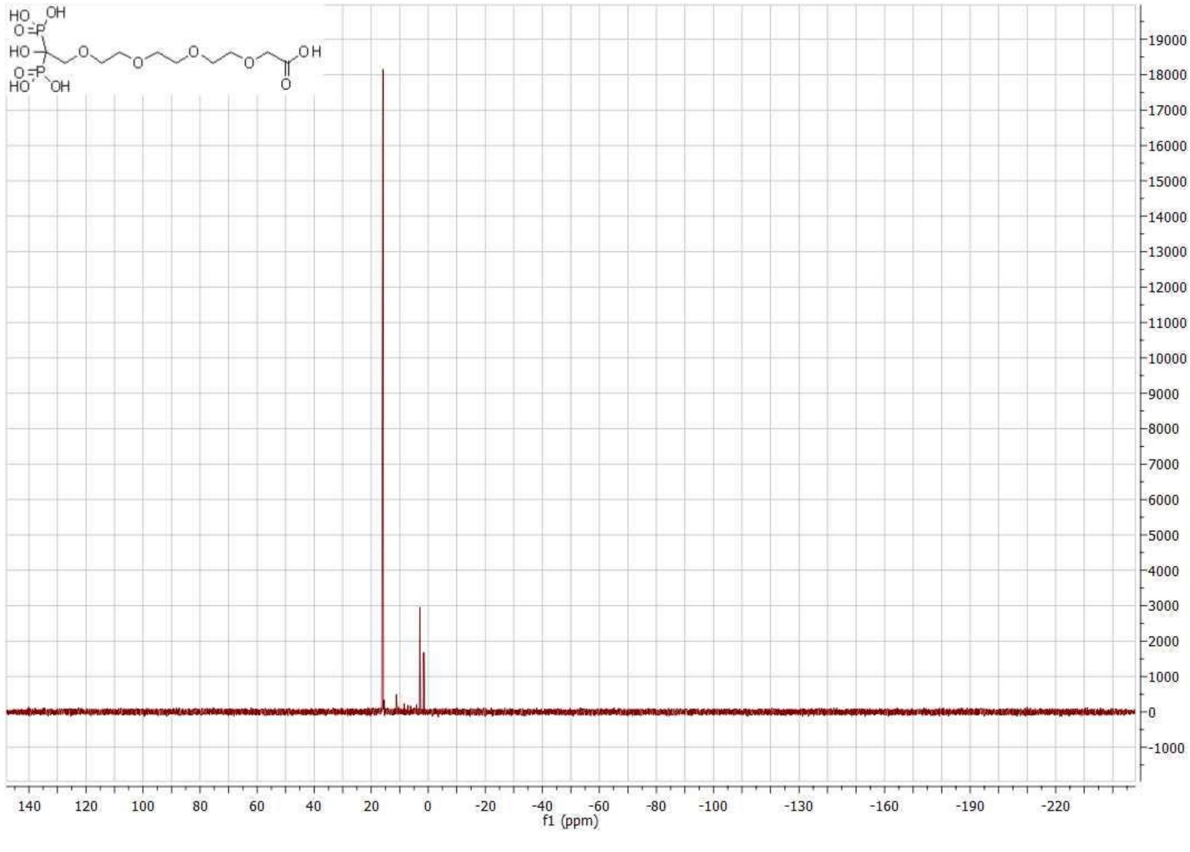

^{31}P NMR spectrum of compound **23** in D_2O

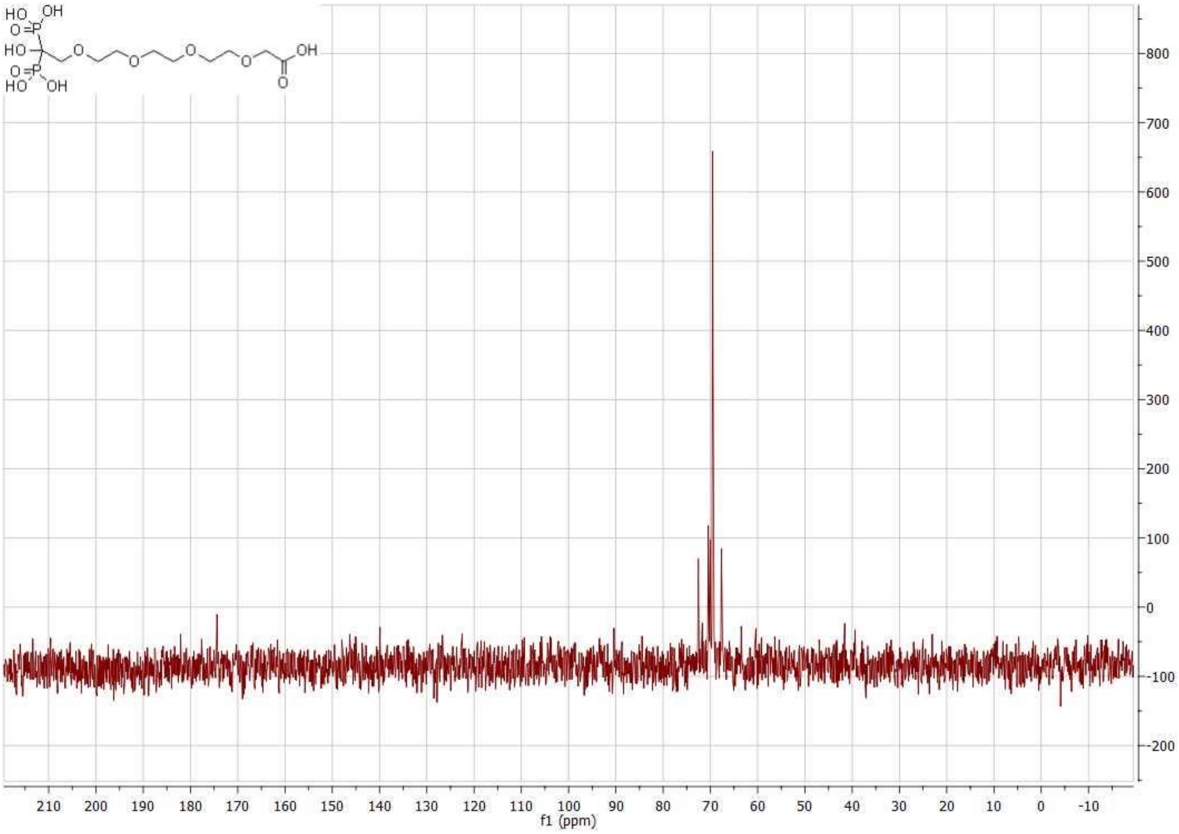

^{13}C NMR spectrum of compound **23** in D_2O
